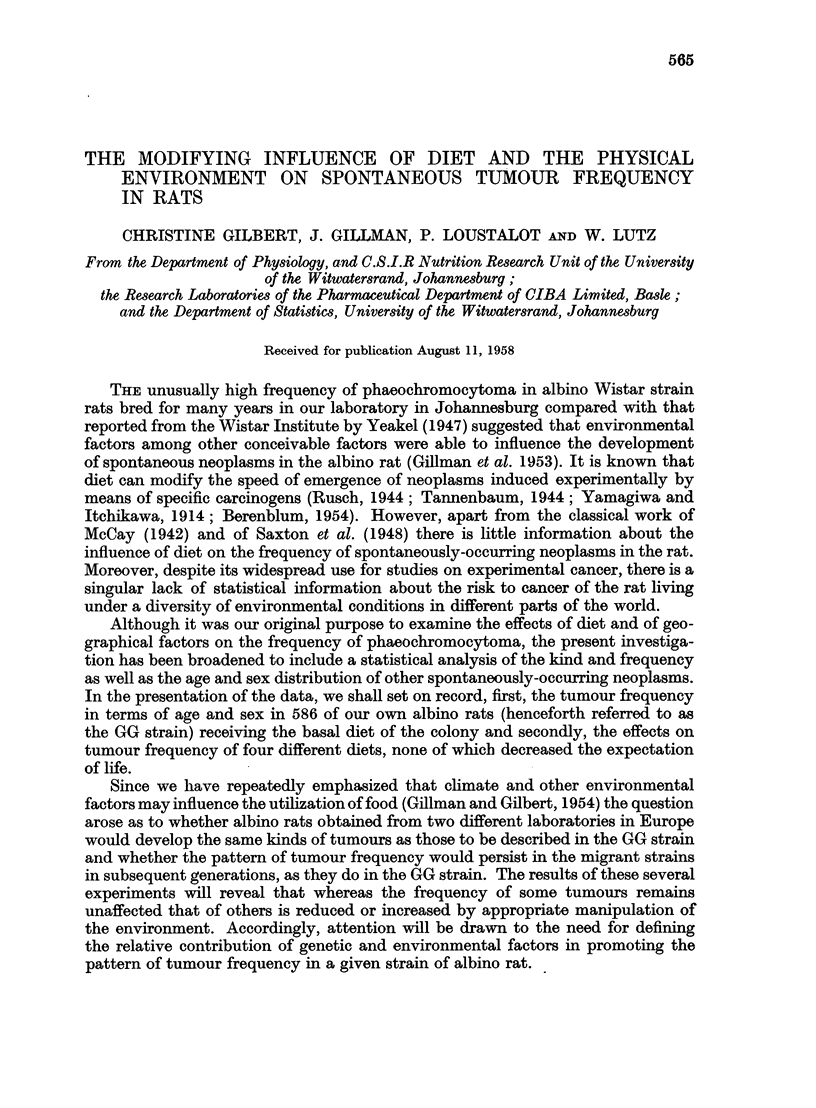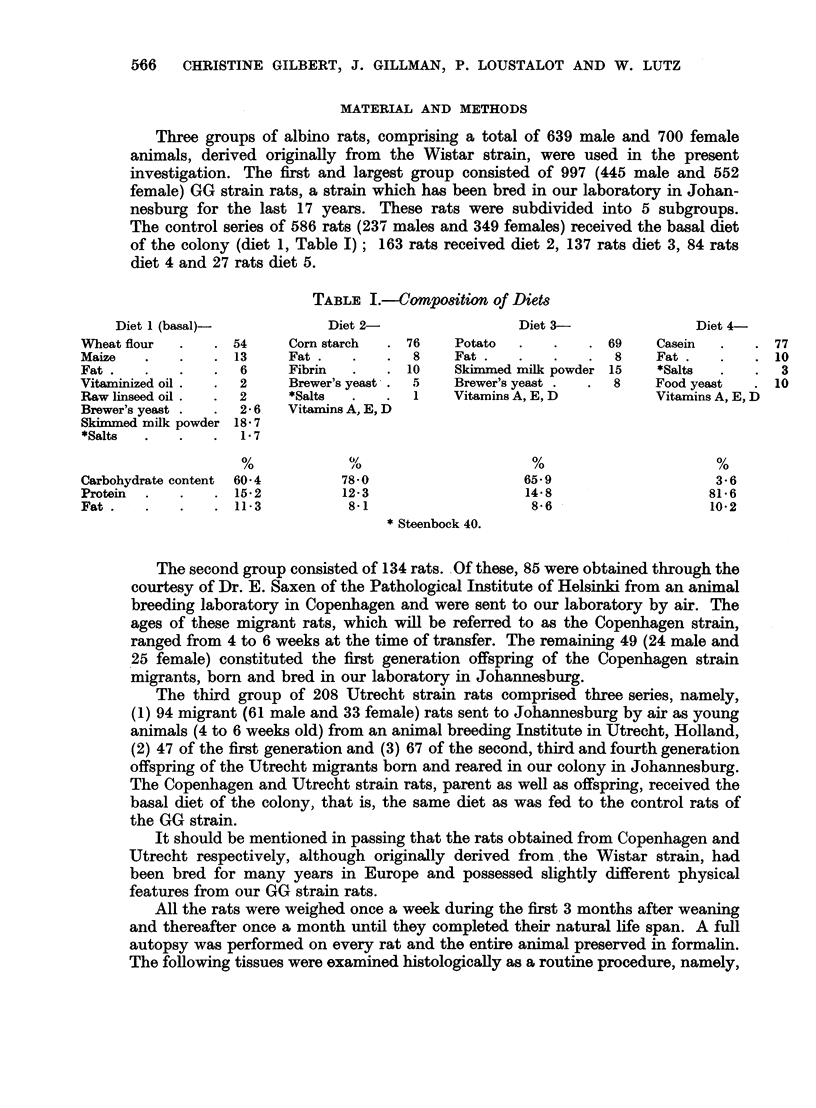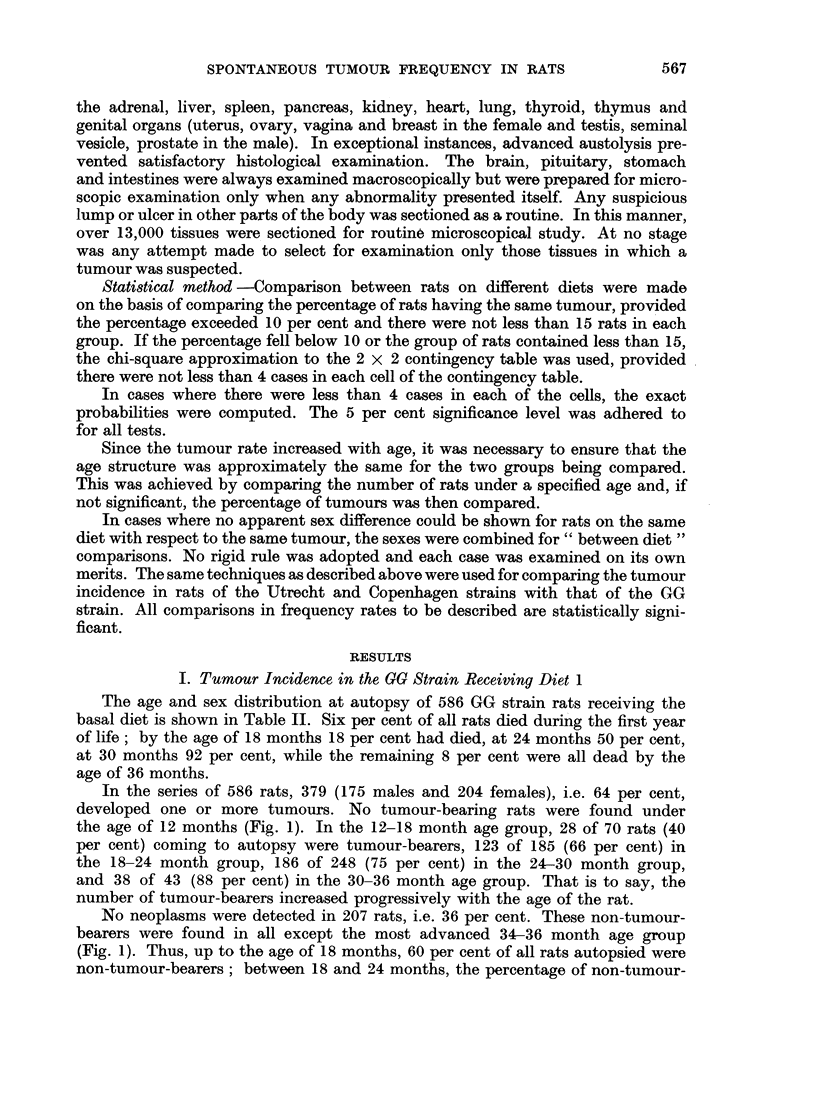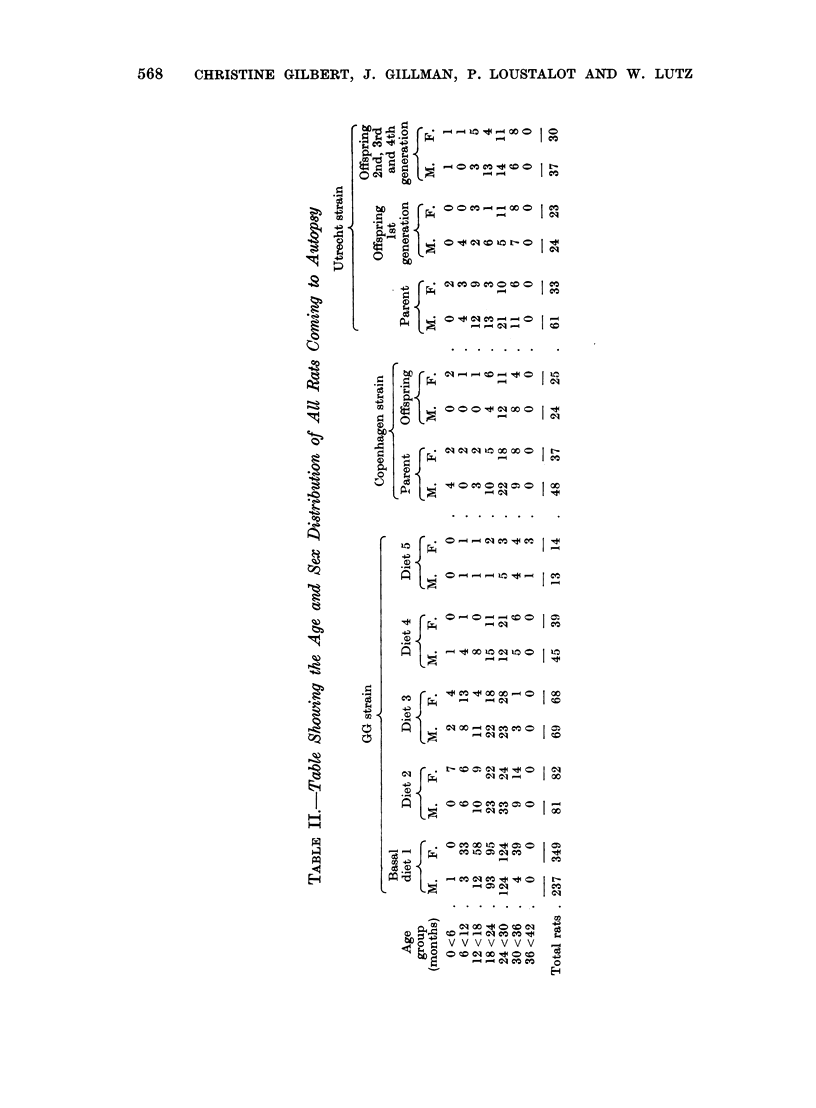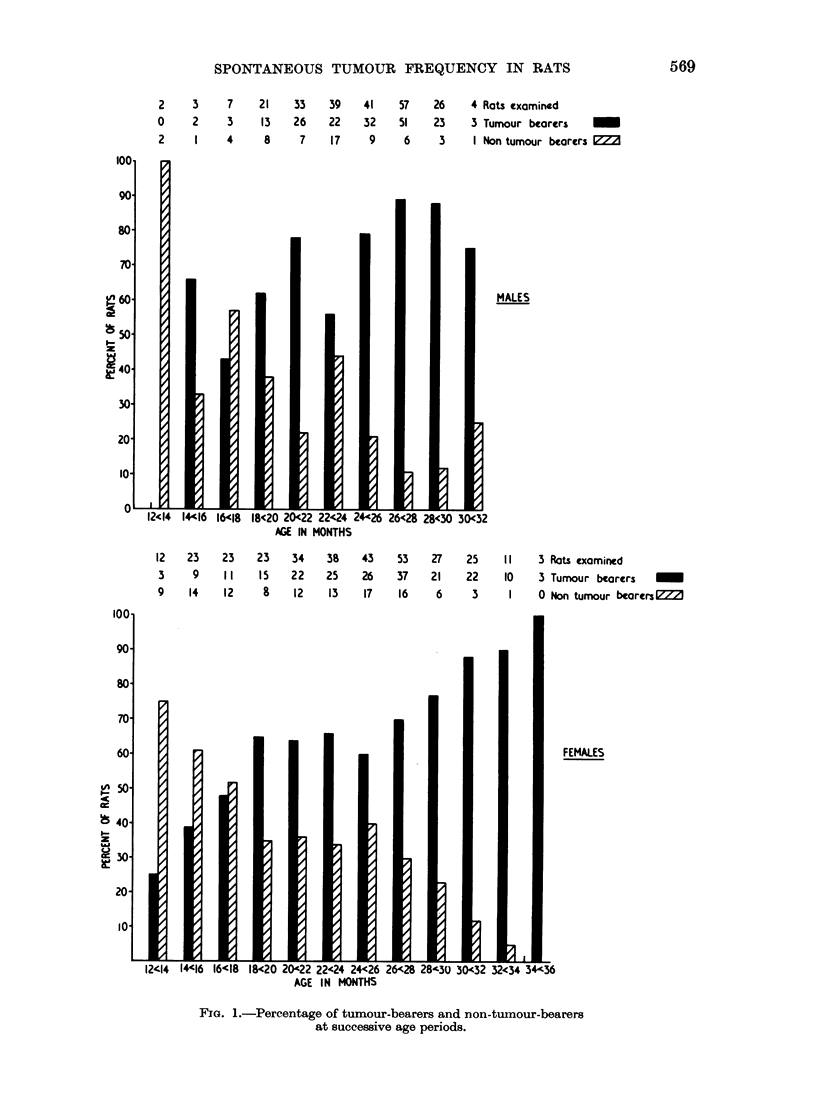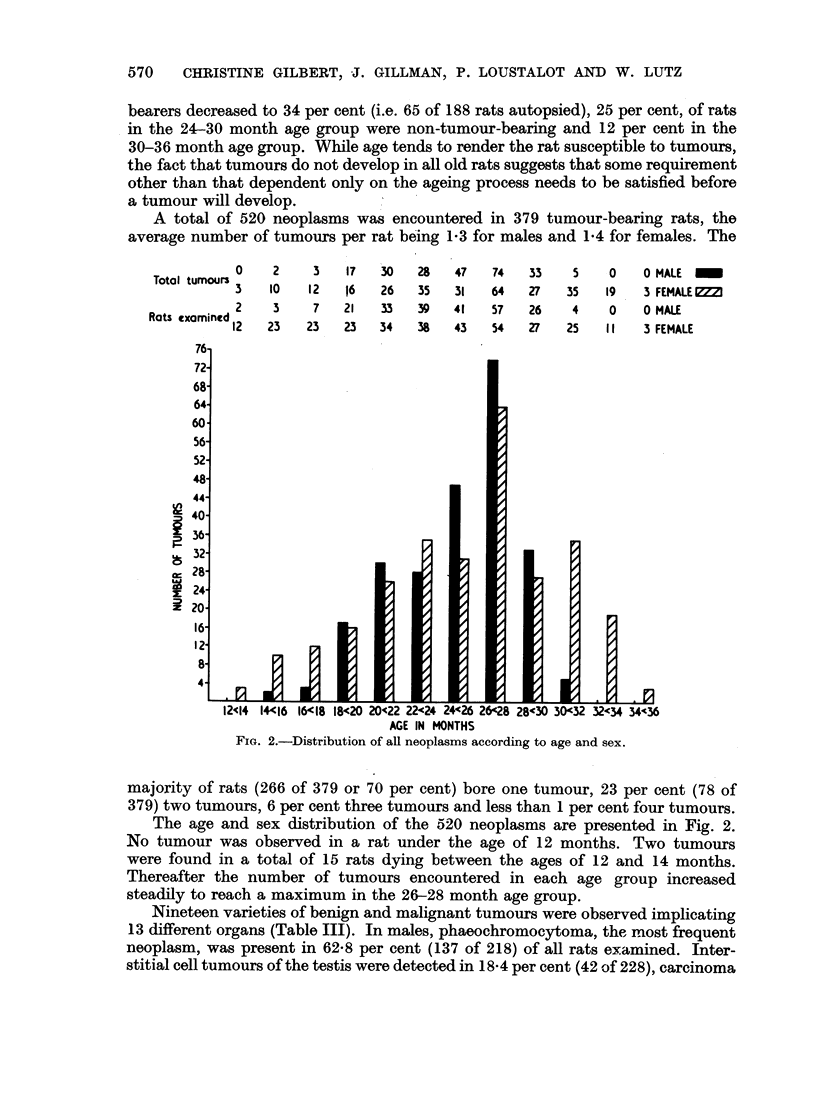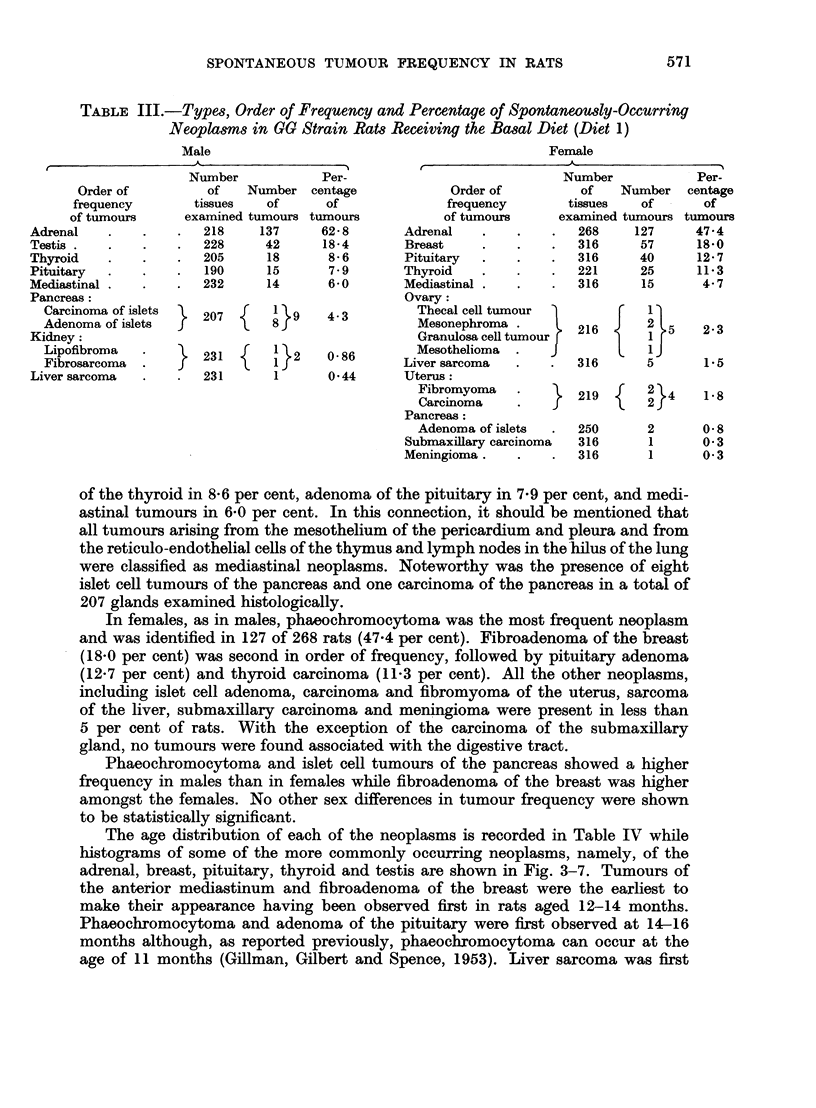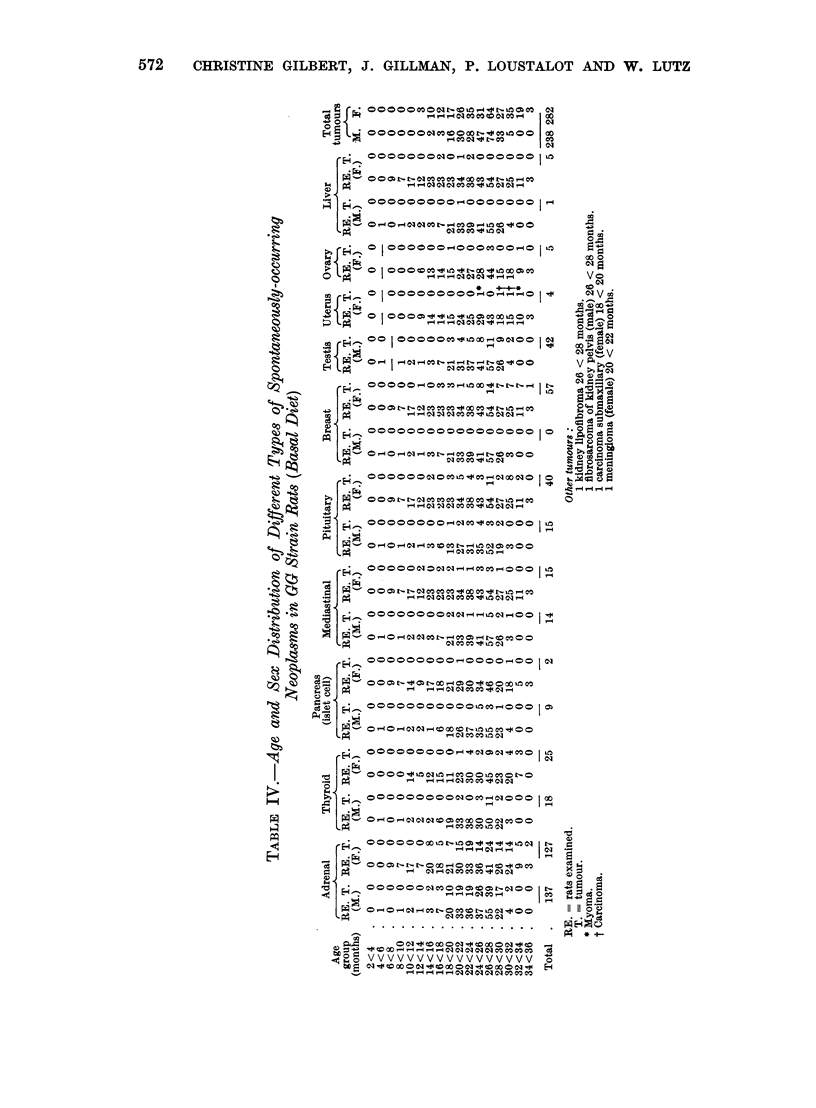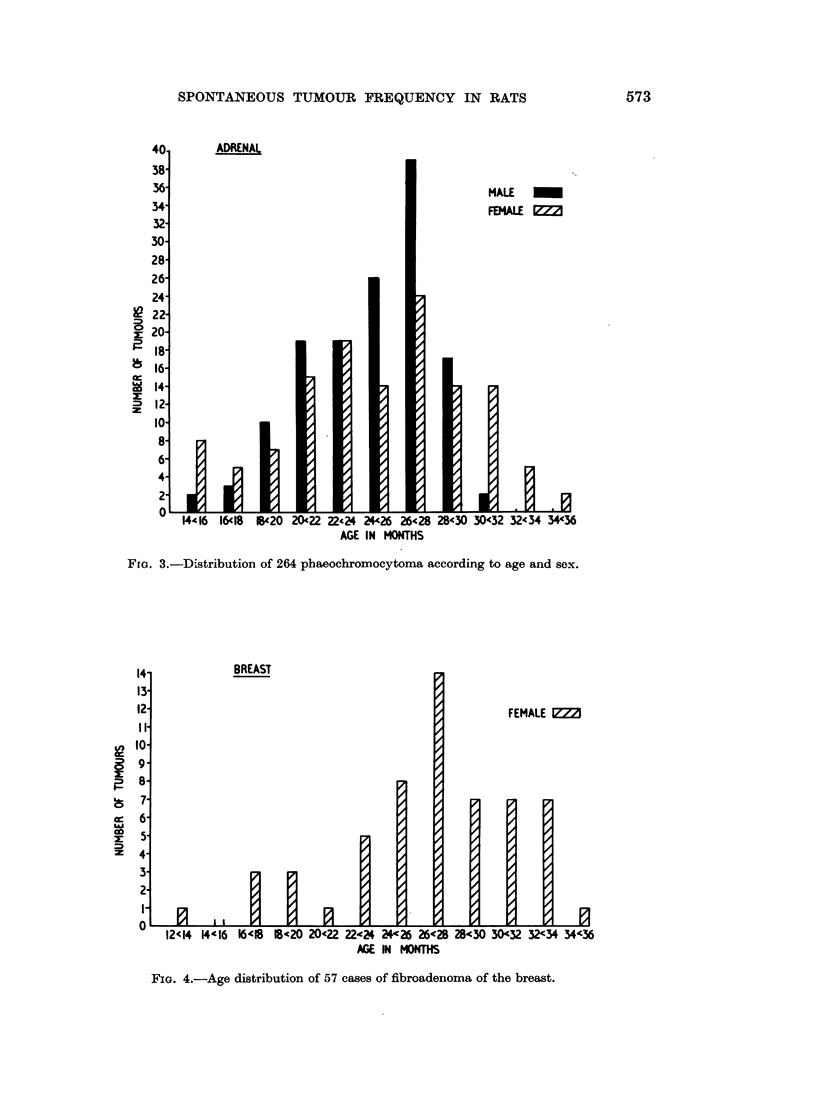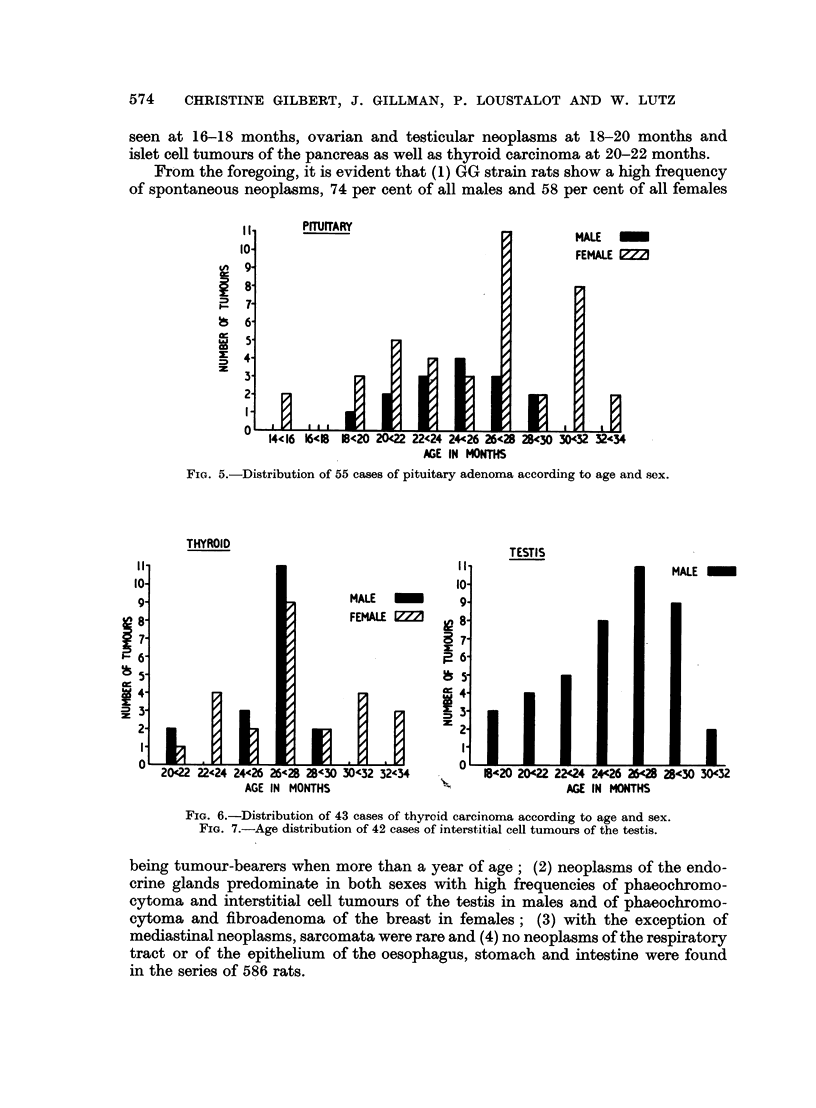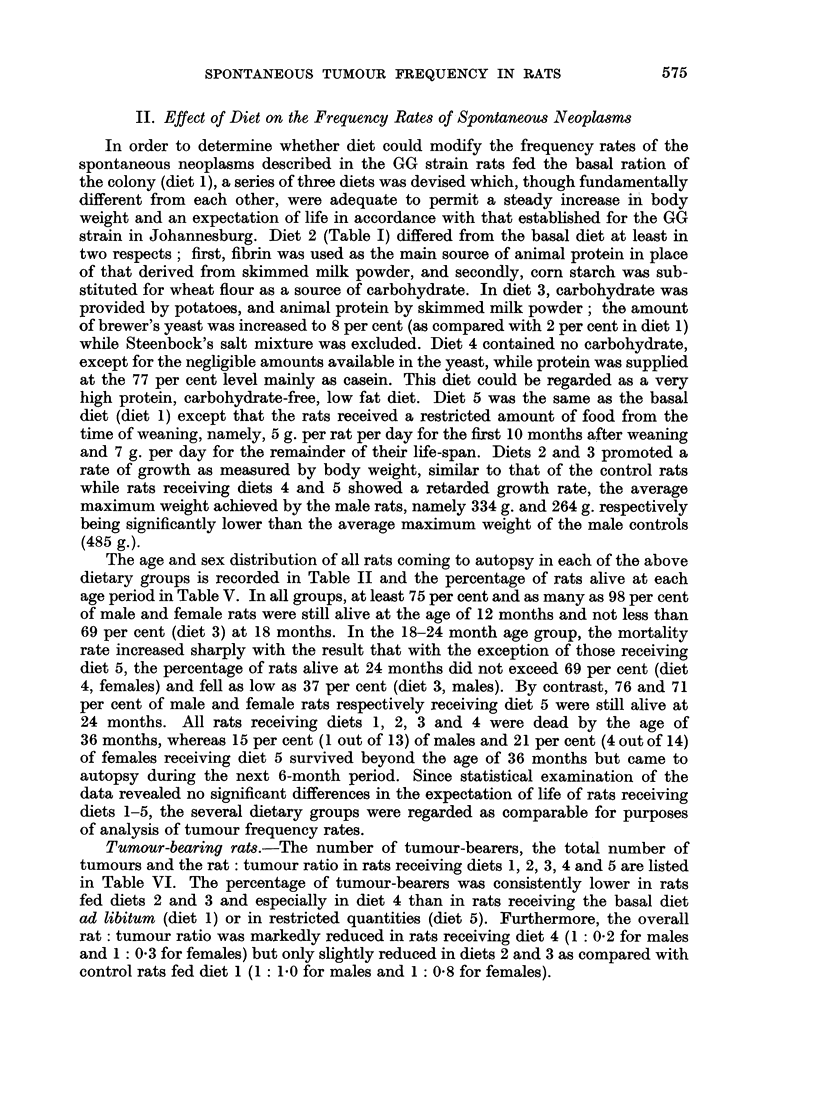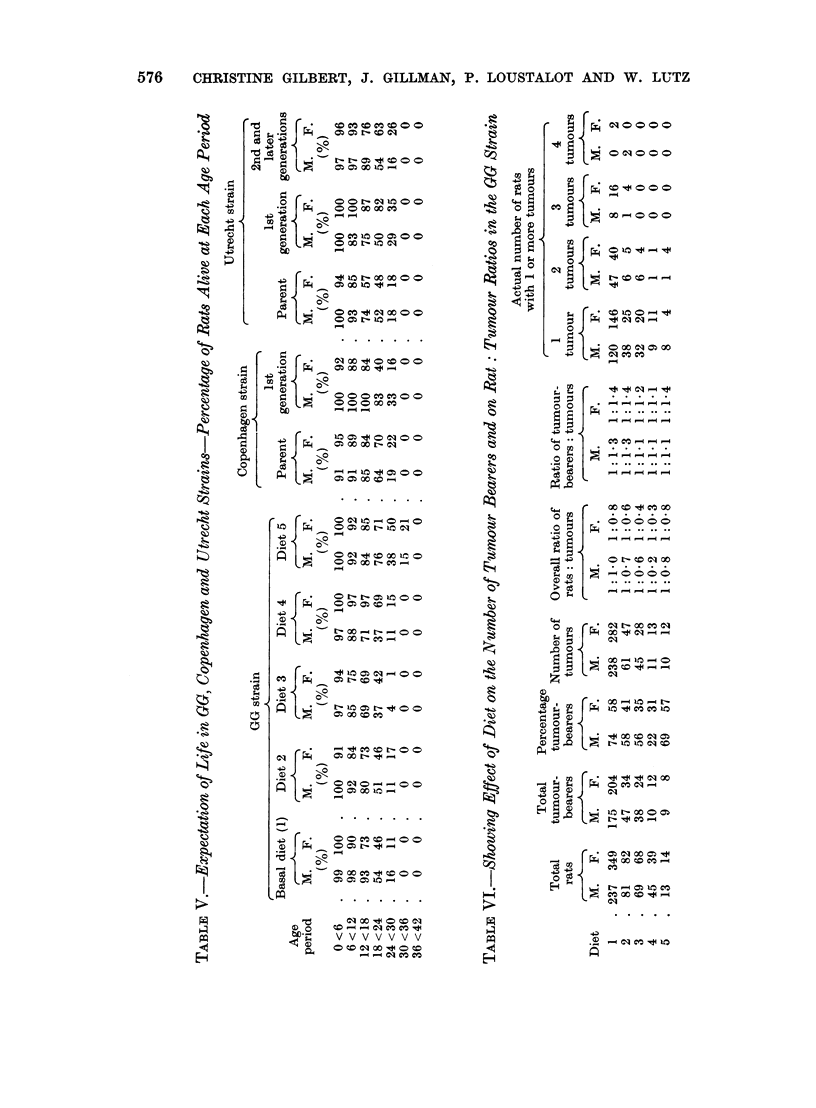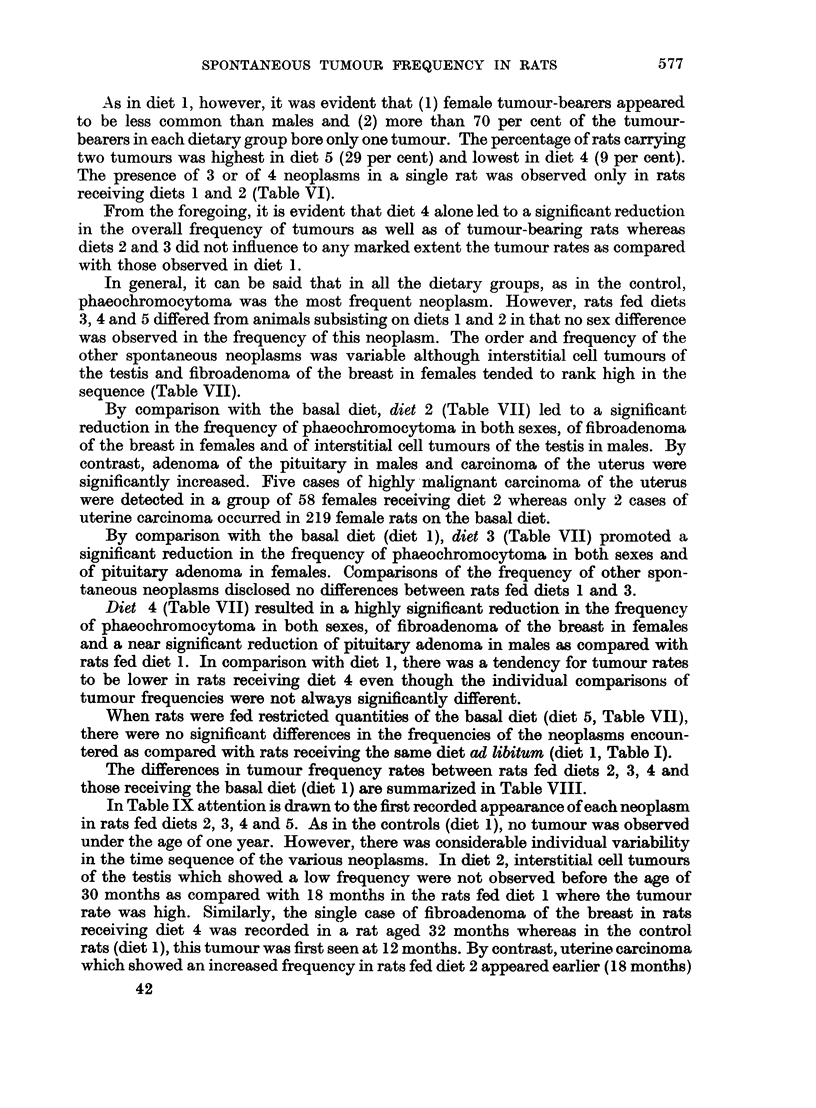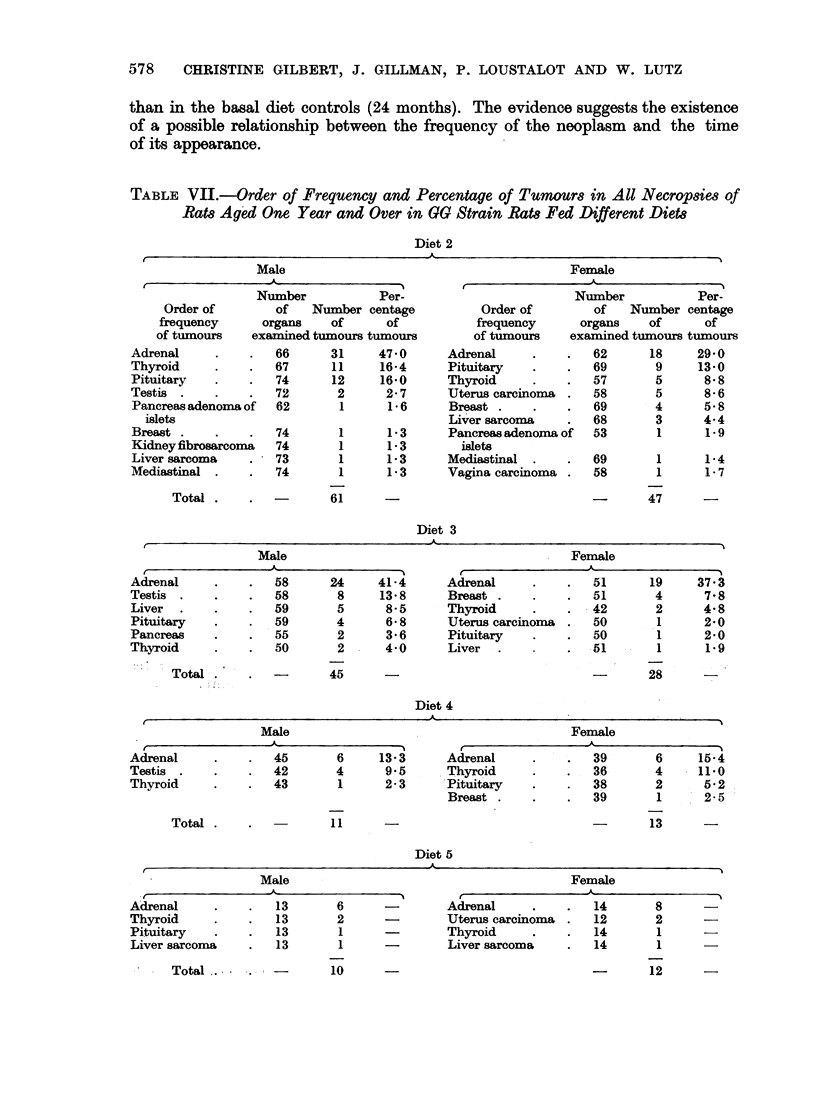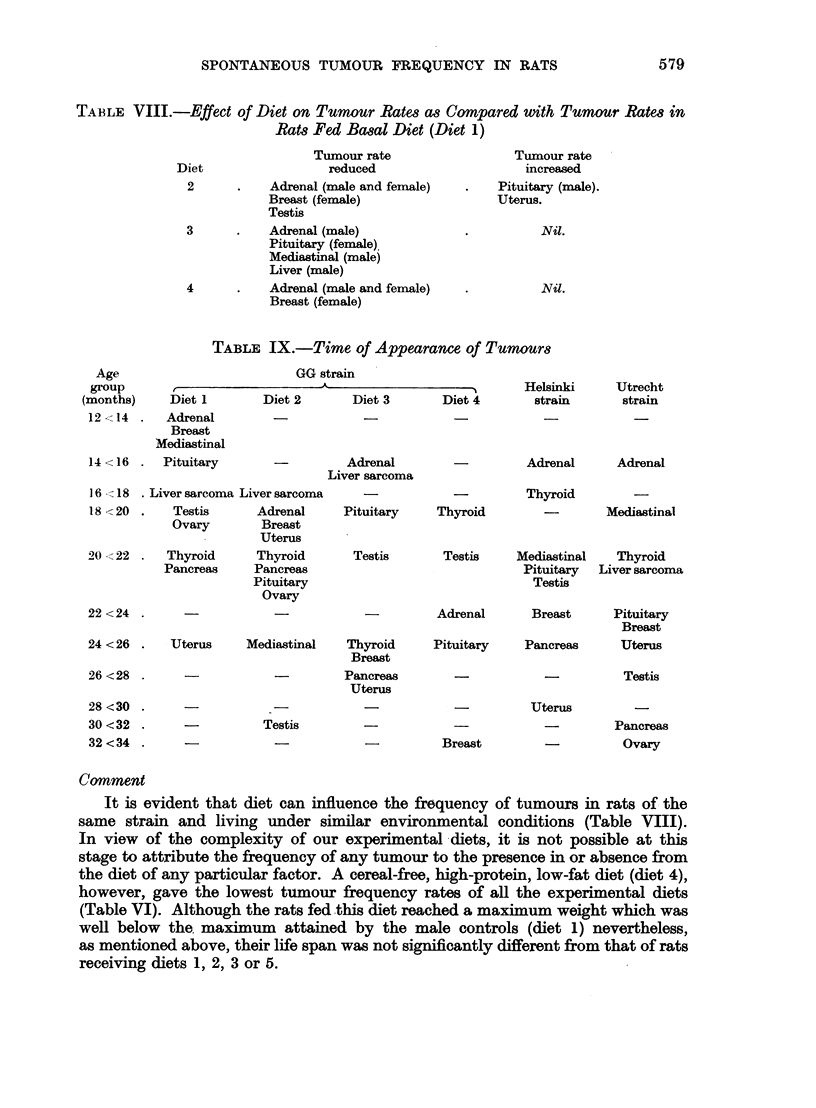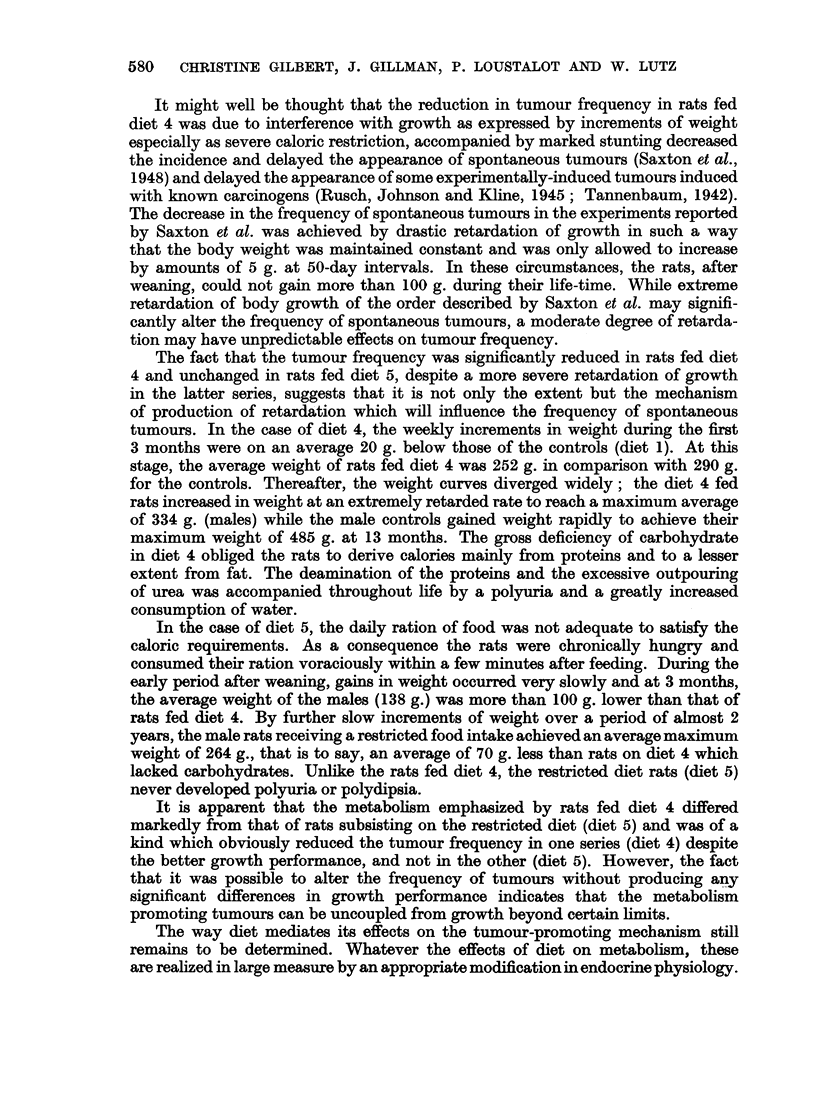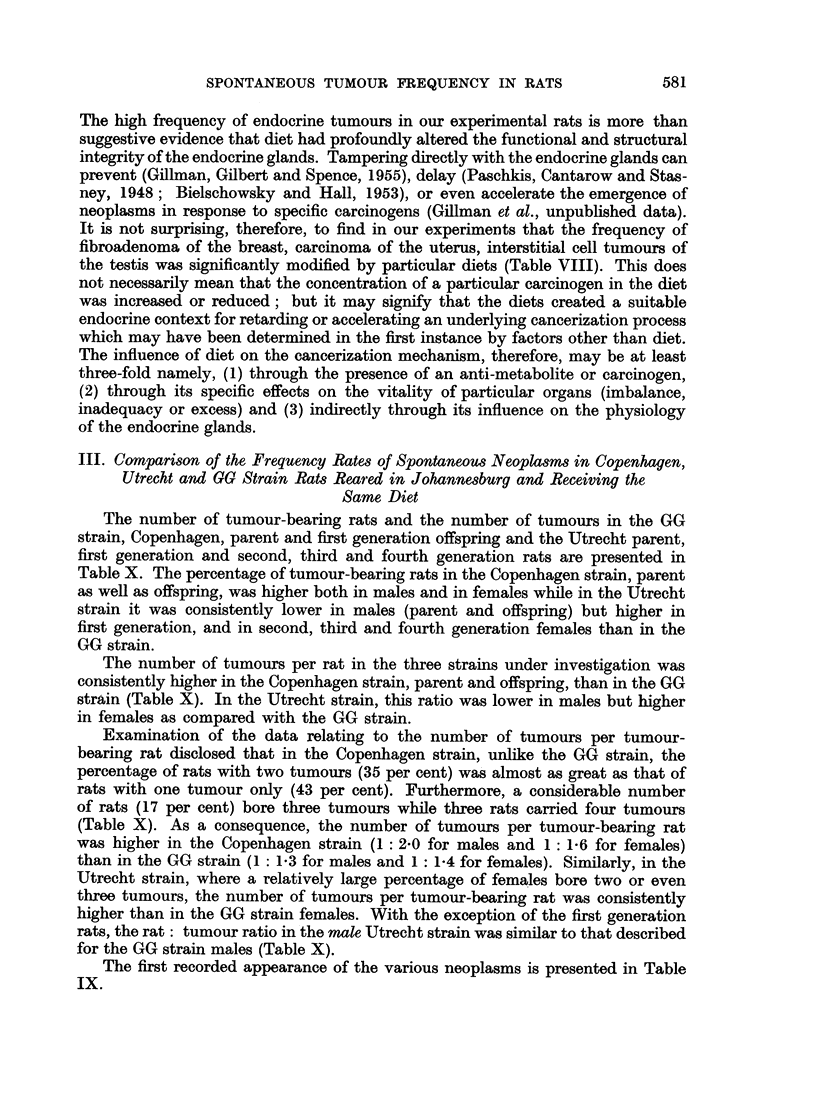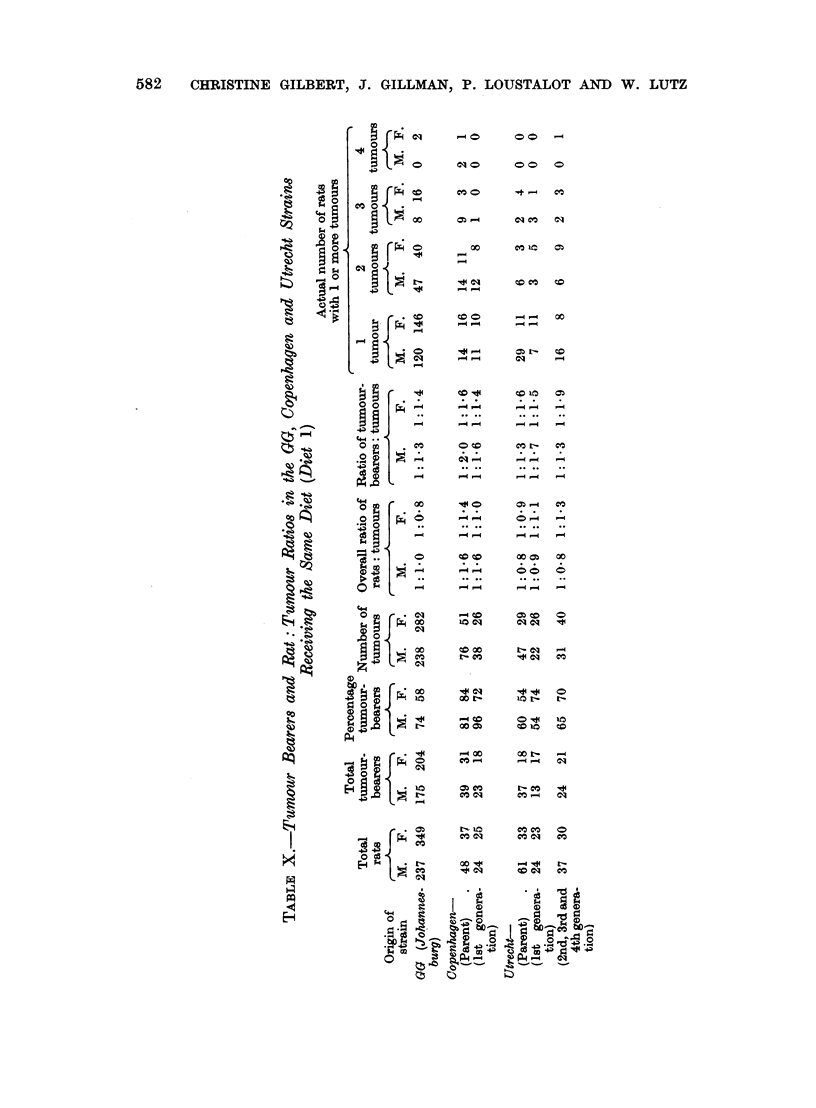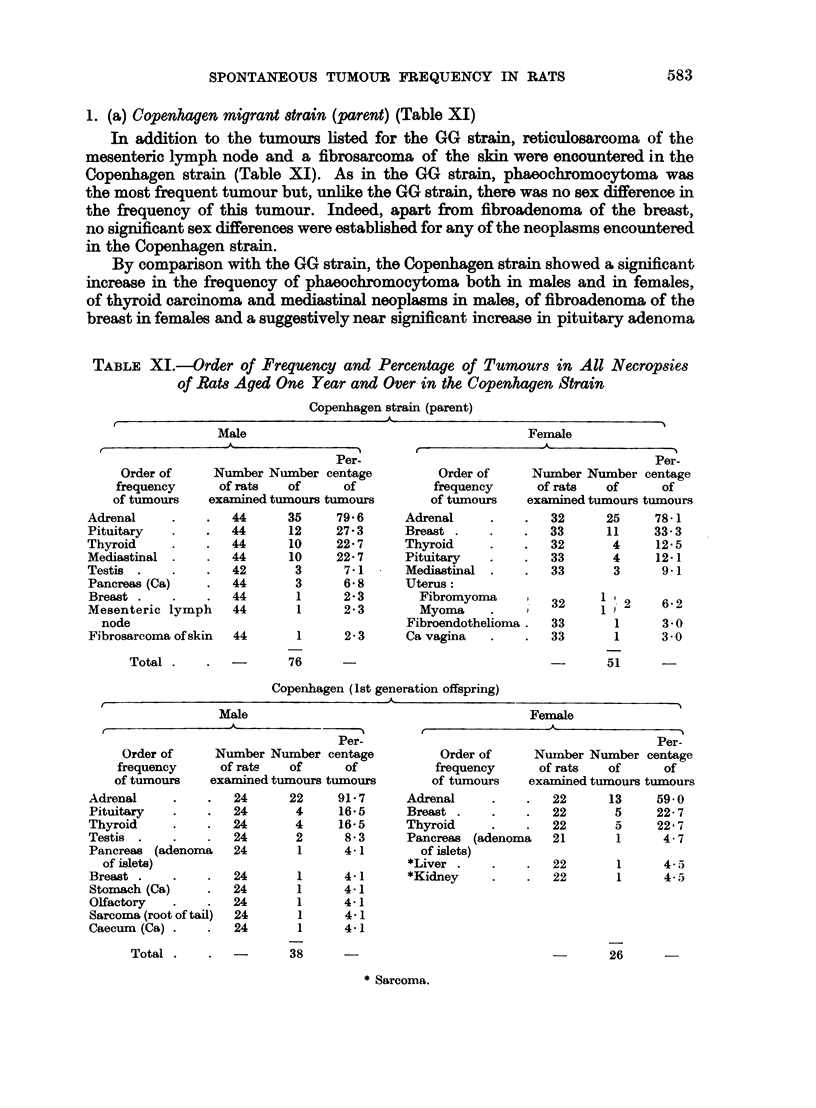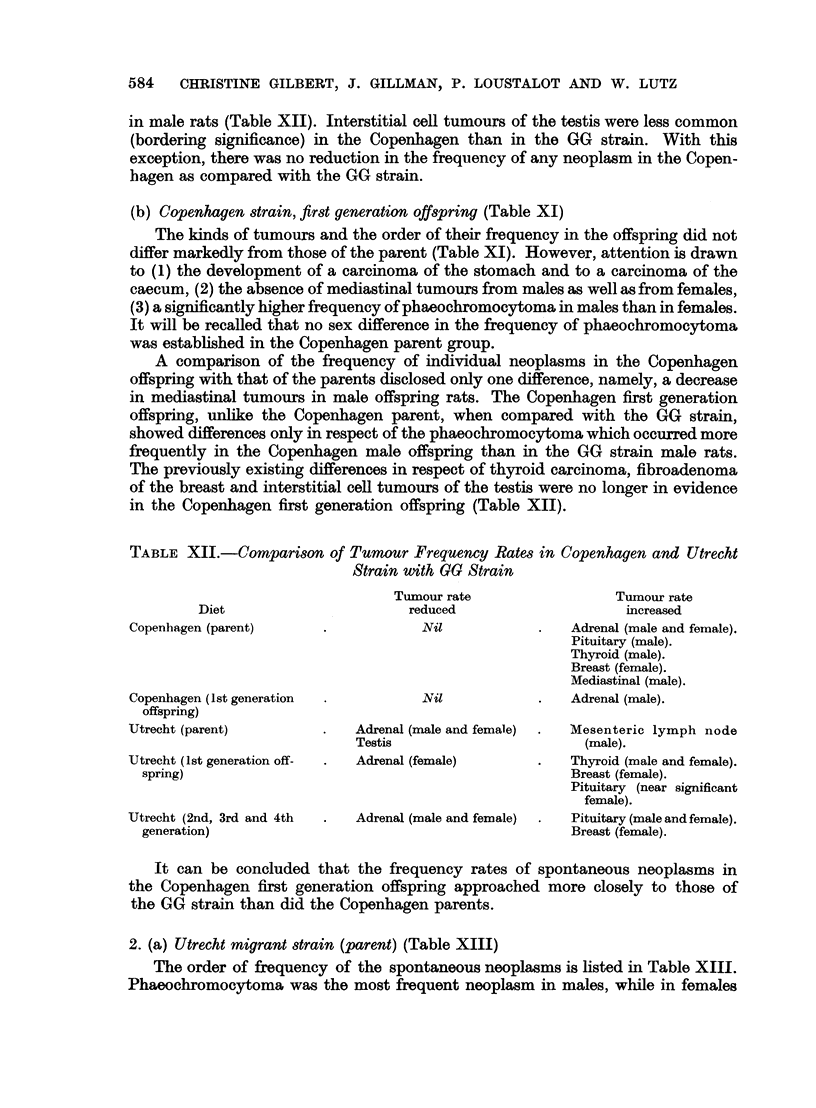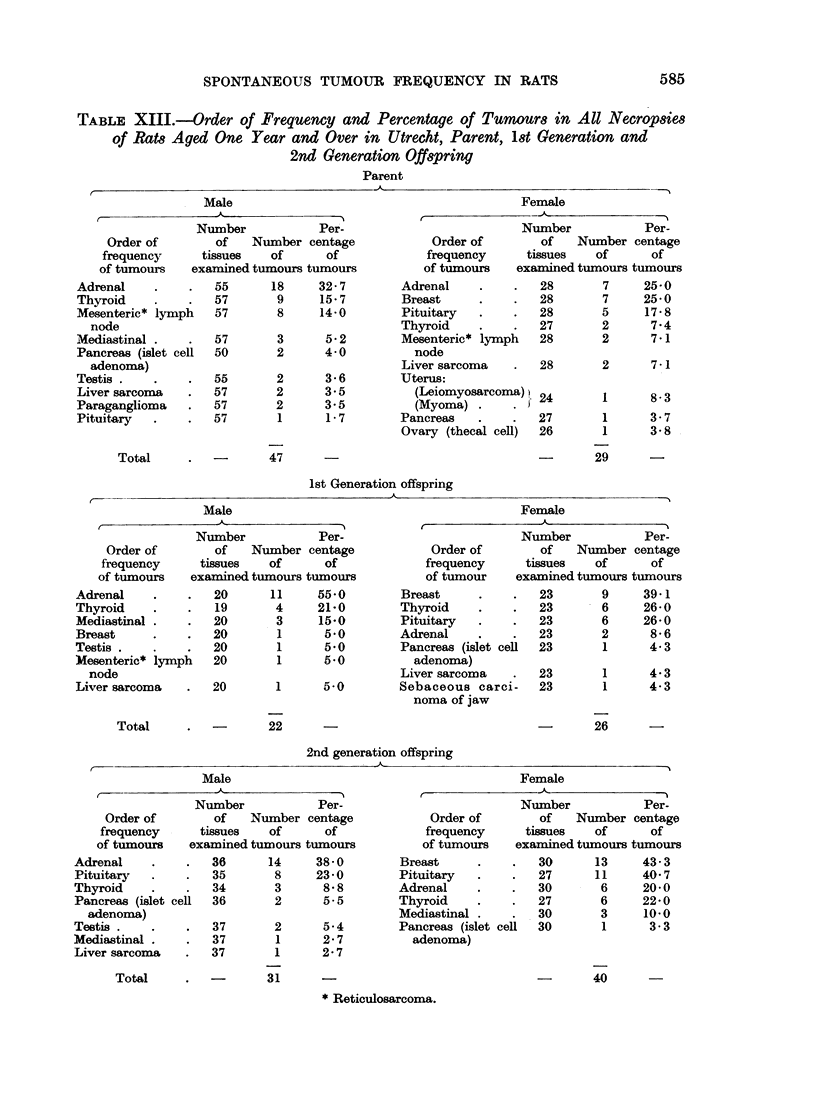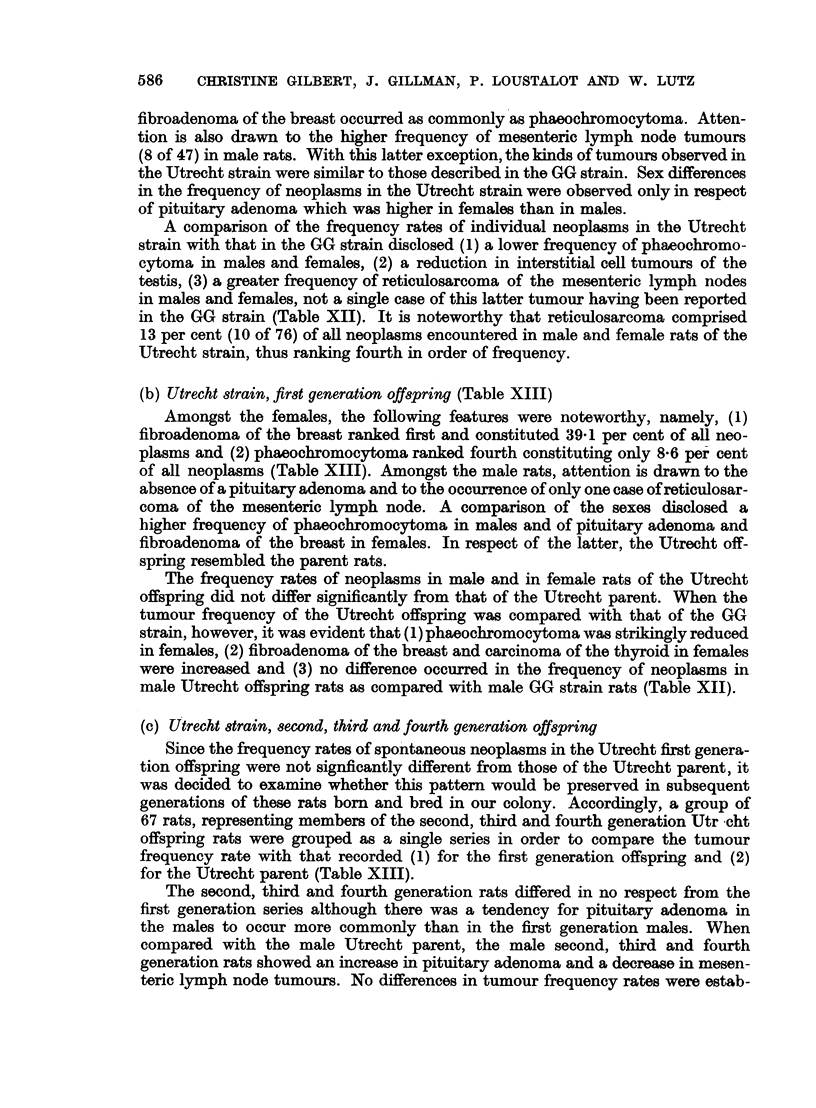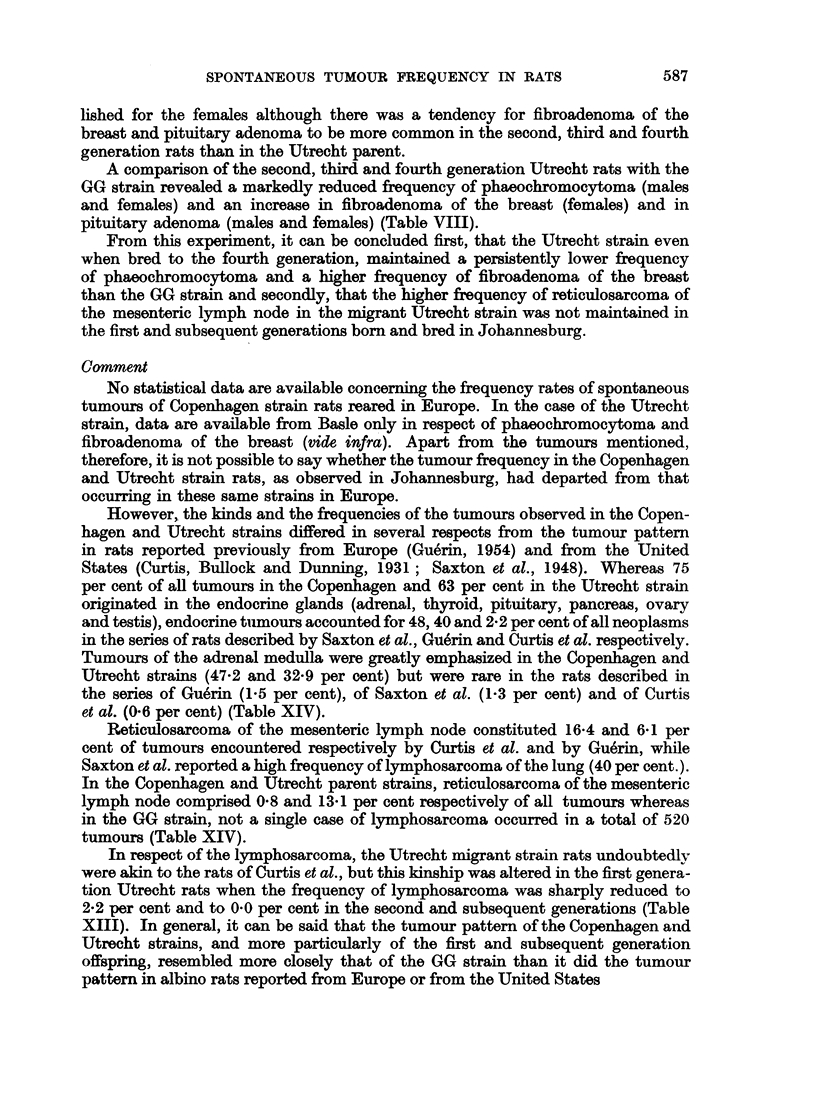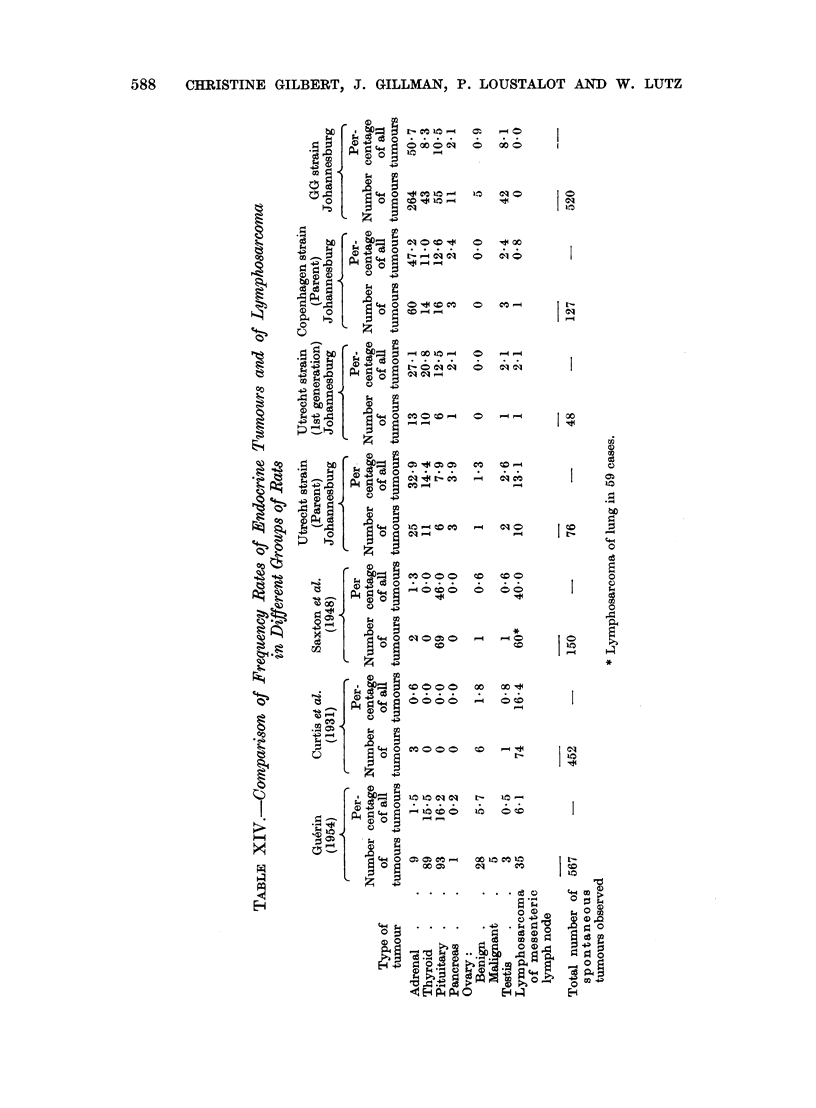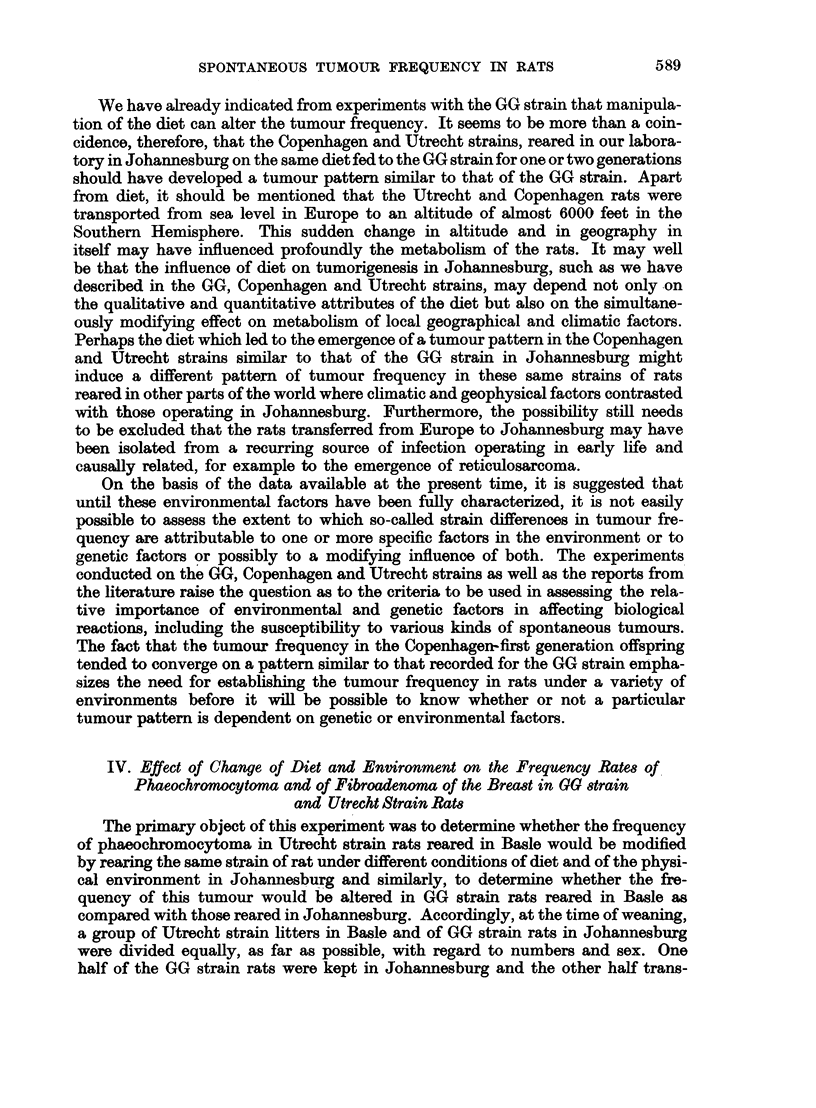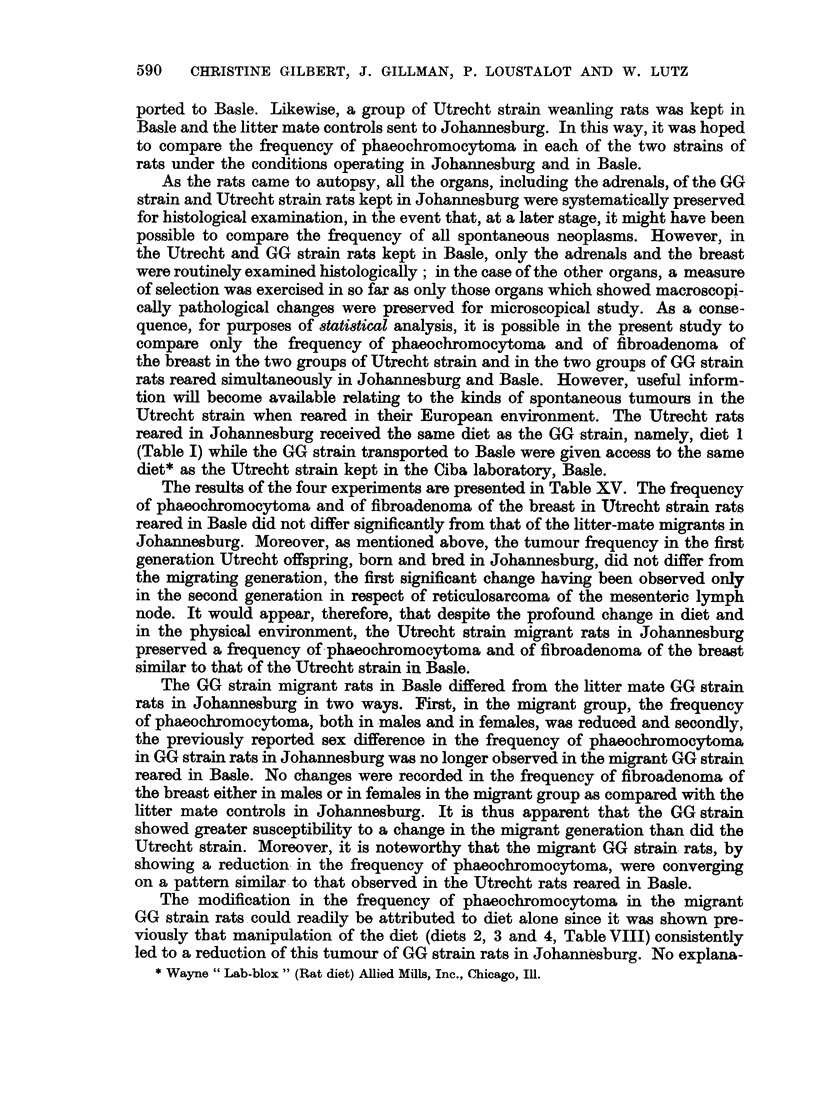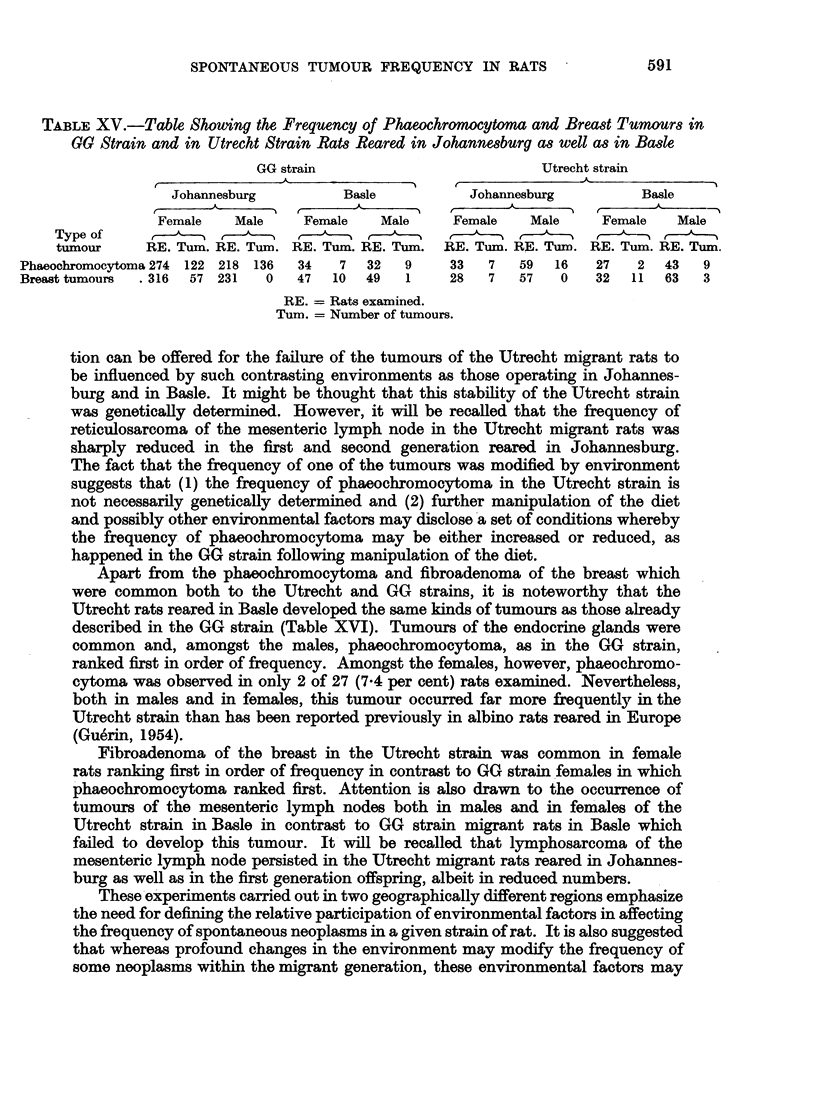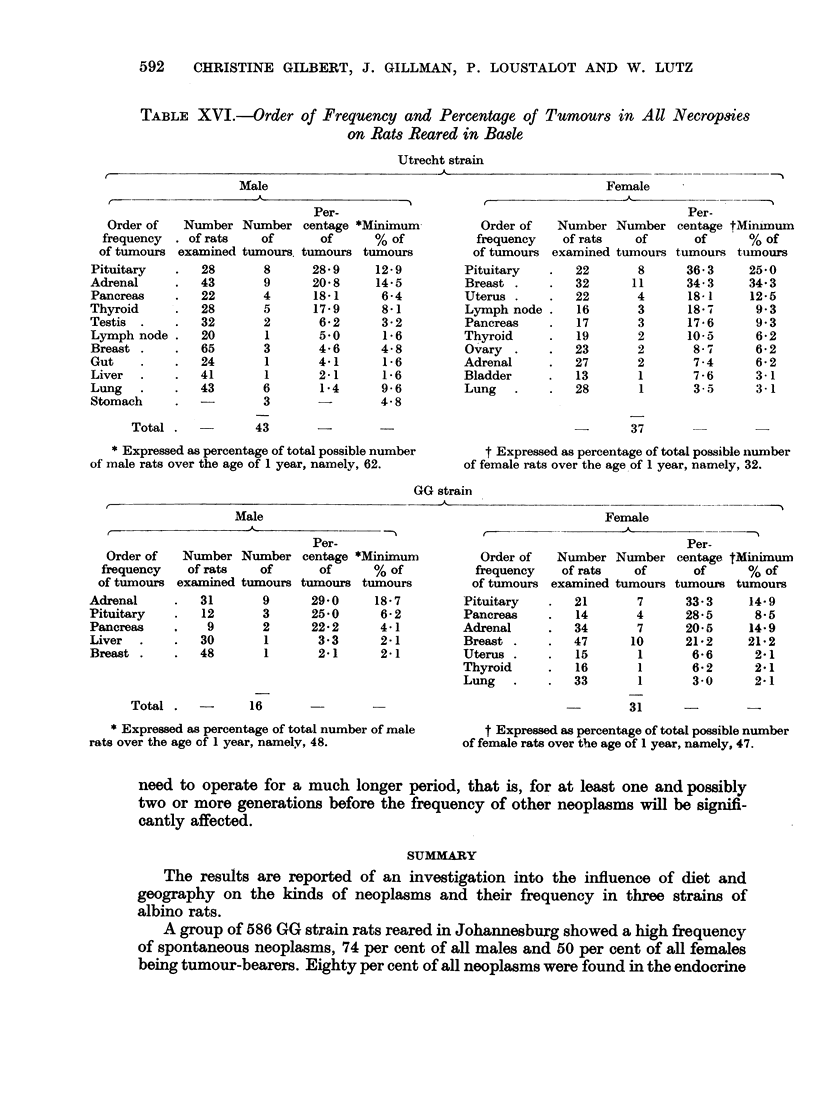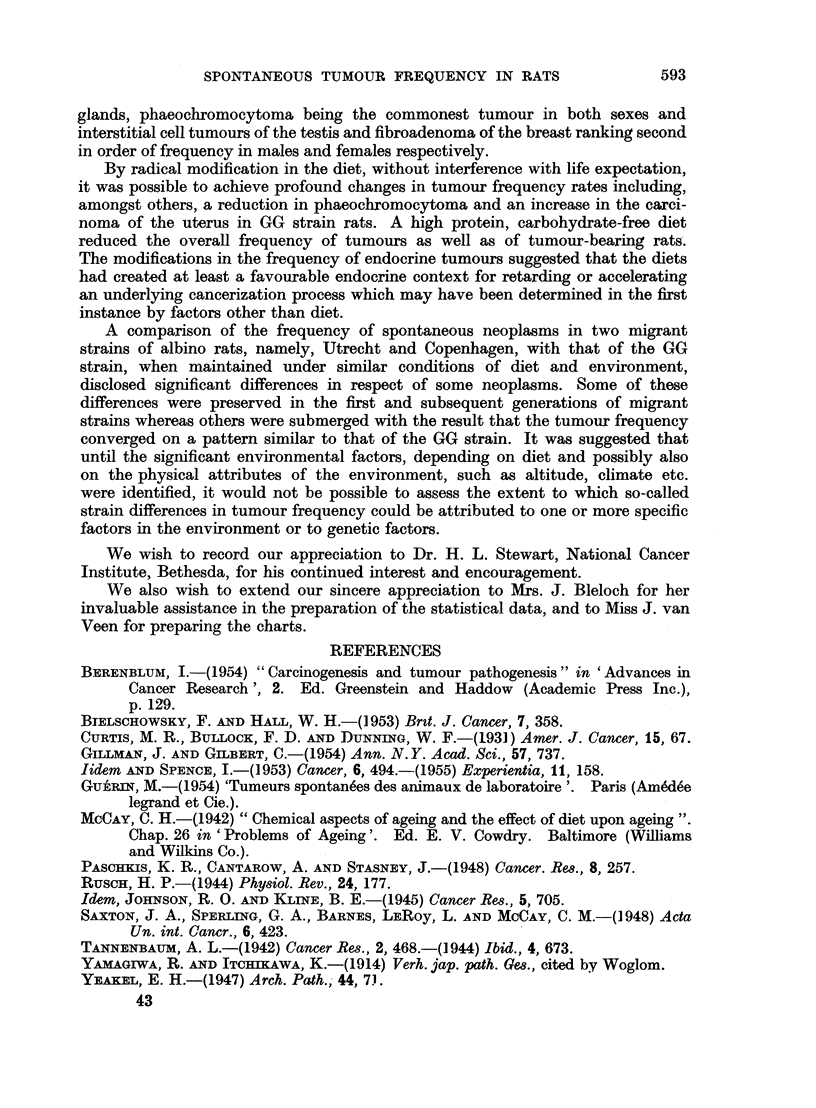# The Modifying Influence of Diet and the Physical Environment on Spontaneous Tumour Frequency in Rats

**DOI:** 10.1038/bjc.1958.66

**Published:** 1958-12

**Authors:** Christine Gilbert, J. Gillman, P. Loustalot, W. Lutz


### THE MODIFYING INFLUENCE OF DIET AND THE PHYSICAL ENVIRONMENT ON SPONTANEOUS TUMOUR FREQUENCY IN RATS

CHRISTINE GILBERT, J. GILLMAN, P. LOUSTALOT AND W. LUTZ

*From the Department of Physiology, and C.S.I.R Nutrition Research Unit of the University of the Witwatersrand, Johannesburg ;*
*the Research Laboratories of the Pharmaceutical Department of CIBA Limited, Basle ;*
*and the Department of Statistics, University of the Witwatersrand, Johannesburg*

Received for publication August 11, 1958

THE unusually high frequency of phaeochromocytoma in albino Wistar strain rats bred for many years in our laboratory in Johannesburg compared with that reported from the Wistar Institute by Yeakel (1947) suggested that environmental factors among other conceivable factors were able to influence the development of spontaneous neoplasms in the albino rat (Gillman *et al.* 1953). It is known that diet can modify the speed of emergence of neoplasms induced experimentally by means of specific carcinogens (Rusch, 1944 ; Tannenbaum, 1944 ; Yamagiwa and Itchikawa, 1914 ; Berenblum, 1954). However, apart from the classical work of McCay (1942) and of Saxton *et al.* (1948) there is little information about the influence of diet on the frequency of spontaneously-occurring neoplasms in the rat. Moreover, despite its widespread use for studies on experimental cancer, there is a singular lack of statistical information about the risk to cancer of the rat living under a diversity of environmental conditions in different parts of the world.

Although it was our original purpose to examine the effects of diet and of geographical factors on the frequency of phaeochromocytoma, the present investigation has been broadened to include a statistical analysis of the kind and frequency as well as the age and sex distribution of other spontaneously-occurring neoplasms. In the presentation of the data, we shall set on record, first, the tumour frequency in terms of age and sex in 586 of our own albino rats (henceforth referred to as the GG strain) receiving the basal diet of the colony and secondly, the effects on tumour frequency of four different diets, none of which decreased the expectation of life.

Since we have repeatedly emphasized that climate and other environmental factors may influence the utilization of food (Gillman and Gilbert, 1954) the question arose as to whether albino rats obtained from two different laboratories in Europe would develop the same kinds of tumours as those to be described in the GG strain and whether the pattern of tumour frequency would persist in the migrant strains in subsequent generations, as they do in the GG strain. The results of these several experiments will reveal that whereas the frequency of some tumours remains unaffected that of others is reduced or increased by appropriate manipulation of the environment. Accordingly, attention will be drawn to the need for defining the relative contribution of genetic and environmental factors in promoting the pattern of tumour frequency in a given strain of albino rat.

### MATERIAL AND METHODS

Three groups of albino rats, comprising a total of 639 male and 700 female animals, derived originally from the Wistar strain, were used in the present investigation. The first and largest group consisted of 997 (445 male and 552 female) GG strain rats, a strain which has been bred in our laboratory in Johannesburg for the last 17 years. These rats were subdivided into 5 subgroups. The control series of 586 rats (237 males and 349 females) received the basal diet of the colony (diet 1, Table I); 163 rats received diet 2, 137 rats diet 3, 84 rats diet 4 and 27 rats diet 5.

TABLE I.—*Composition of Diets*

| Diet 1 (basal)— | | Diet 2— | | Diet 3— | | Diet 4— | |
|---|---|---|---|---|---|---|---|
| Wheat flour | 54 | Corn starch | 76 | Potato | 69 | Casein | 77 |
| Maize | 13 | Fat | 8 | Fat | 8 | Fat | 10 |
| Fat | 6 | Fibrin | 10 | Skimmed milk powder | 15 | *Salts | 3 |
| Vitaminized oil | 2 | Brewer's yeast | 5 | Brewer's yeast | 8 | Food yeast | 10 |
| Raw linseed oil | 2 | *Salts | 1 | Vitamins A, E, D | | Vitamins A, E, D | |
| Brewer's yeast | 2·6 | Vitamins A, E, D | | | | | |
| Skimmed milk powder | 18·7 | | | | | | |
| *Salts | 1·7 | | | | | | |
| | % | | % | | % | | % |
| Carbohydrate content | 60·4 | | 78·0 | | 65·9 | | 3·6 |
| Protein | 15·2 | | 12·3 | | 14·8 | | 81·6 |
| Fat | 11·3 | | 8·1 | | 8·6 | | 10·2 |

* Steenbock 40.

The second group consisted of 134 rats. Of these, 85 were obtained through the courtesy of Dr. E. Saxen of the Pathological Institute of Helsinki from an animal breeding laboratory in Copenhagen and were sent to our laboratory by air. The ages of these migrant rats, which will be referred to as the Copenhagen strain, ranged from 4 to 6 weeks at the time of transfer. The remaining 49 (24 male and 25 female) constituted the first generation offspring of the Copenhagen strain migrants, born and bred in our laboratory in Johannesburg.

The third group of 208 Utrecht strain rats comprised three series, namely, (1) 94 migrant (61 male and 33 female) rats sent to Johannesburg by air as young animals (4 to 6 weeks old) from an animal breeding Institute in Utrecht, Holland, (2) 47 of the first generation and (3) 67 of the second, third and fourth generation offspring of the Utrecht migrants born and reared in our colony in Johannesburg. The Copenhagen and Utrecht strain rats, parent as well as offspring, received the basal diet of the colony, that is, the same diet as was fed to the control rats of the GG strain.

It should be mentioned in passing that the rats obtained from Copenhagen and Utrecht respectively, although originally derived from the Wistar strain, had been bred for many years in Europe and possessed slightly different physical features from our GG strain rats.

All the rats were weighed once a week during the first 3 months after weaning and thereafter once a month until they completed their natural life span. A full autopsy was performed on every rat and the entire animal preserved in formalin. The following tissues were examined histologically as a routine procedure, namely, the adrenal, liver, spleen, pancreas, kidney, heart, lung, thyroid, thymus and genital organs (uterus, ovary, vagina and breast in the female and testis, seminal vesicle, prostate in the male). In exceptional instances, advanced austolysis prevented satisfactory histological examination. The brain, pituitary, stomach and intestines were always examined macroscopically but were prepared for microscopic examination only when any abnormality presented itself. Any suspicious lump or ulcer in other parts of the body was sectioned as a routine. In this manner, over 13,000 tissues were sectioned for routine microscopical study. At no stage was any attempt made to select for examination only those tissues in which a tumour was suspected.

*Statistical method*—Comparison between rats on different diets were made on the basis of comparing the percentage of rats having the same tumour, provided the percentage exceeded 10 per cent and there were not less than 15 rats in each group. If the percentage fell below 10 or the group of rats contained less than 15, the chi-square approximation to the $2 \times 2$ contingency table was used, provided there were not less than 4 cases in each cell of the contingency table.

In cases where there were less than 4 cases in each of the cells, the exact probabilities were computed. The 5 per cent significance level was adhered to for all tests.

Since the tumour rate increased with age, it was necessary to ensure that the age structure was approximately the same for the two groups being compared. This was achieved by comparing the number of rats under a specified age and, if not significant, the percentage of tumours was then compared.

In cases where no apparent sex difference could be shown for rats on the same diet with respect to the same tumour, the sexes were combined for " between diet " comparisons. No rigid rule was adopted and each case was examined on its own merits. The same techniques as described above were used for comparing the tumour incidence in rats of the Utrecht and Copenhagen strains with that of the GG strain. All comparisons in frequency rates to be described are statistically significant.

### RESULTS

### I. *Tumour Incidence in the GG Strain Receiving Diet* 1

The age and sex distribution at autopsy of 586 GG strain rats receiving the basal diet is shown in Table II. Six per cent of all rats died during the first year of life ; by the age of 18 months 18 per cent had died, at 24 months 50 per cent, at 30 months 92 per cent, while the remaining 8 per cent were all dead by the age of 36 months.

In the series of 586 rats, 379 (175 males and 204 females), i.e. 64 per cent, developed one or more tumours. No tumour-bearing rats were found under the age of 12 months (Fig. 1). In the 12–18 month age group, 28 of 70 rats (40 per cent) coming to autopsy were tumour-bearers, 123 of 185 (66 per cent) in the 18–24 month group, 186 of 248 (75 per cent) in the 24–30 month group, and 38 of 43 (88 per cent) in the 30–36 month age group. That is to say, the number of tumour-bearers increased progressively with the age of the rat.

No neoplasms were detected in 207 rats, i.e. 36 per cent. These non-tumour-bearers were found in all except the most advanced 34–36 month age group (Fig. 1). Thus, up to the age of 18 months, 60 per cent of all rats autopsied were non-tumour-bearers ; between 18 and 24 months, the percentage of non-tumour-

TABLE II.—*Table Showing the Age and Sex Distribution of All Rats Coming to Autopsy*

| Age group (months) | GG strain: Basal diet 1 M. | GG strain: Basal diet 1 F. | Diet 2 M. | Diet 2 F. | Diet 3 M. | Diet 3 F. | Diet 4 M. | Diet 4 F. | Diet 5 M. | Diet 5 F. | Copenhagen strain: Parent M. | Parent F. | Offspring M. | Offspring F. | Utrecht strain: Parent M. | Parent F. | Offspring 1st generation M. | Offspring 1st generation F. | Offspring 2nd, 3rd and 4th generation M. | Offspring 2nd, 3rd and 4th generation F. |
|---|---|---|---|---|---|---|---|---|---|---|---|---|---|---|---|---|---|---|---|---|
| 0 <6 | 1 | 0 | 0 | 7 | 2 | 4 | 1 | 0 | 0 | 0 | 4 | 2 | 0 | 2 | 0 | 2 | 0 | 0 | 1 | 1 |
| 6 <12 | 3 | 33 | 6 | 6 | 8 | 13 | 4 | 1 | 1 | 1 | 0 | 2 | 0 | 1 | 4 | 3 | 4 | 0 | 0 | 1 |
| 12 <18 | 12 | 58 | 10 | 9 | 11 | 4 | 8 | 0 | 1 | 1 | 3 | 2 | 0 | 1 | 12 | 9 | 2 | 3 | 3 | 5 |
| 18 <24 | 93 | 95 | 23 | 22 | 22 | 18 | 15 | 11 | 1 | 2 | 10 | 5 | 4 | 6 | 13 | 3 | 6 | 1 | 13 | 4 |
| 24 <30 | 124 | 124 | 33 | 24 | 23 | 28 | 12 | 21 | 5 | 3 | 22 | 18 | 12 | 11 | 21 | 10 | 5 | 11 | 14 | 11 |
| 30 <36 | 4 | 39 | 9 | 14 | 3 | 1 | 5 | 6 | 4 | 4 | 9 | 8 | 8 | 4 | 11 | 6 | 7 | 8 | 6 | 8 |
| 36 <42 | 0 | 0 | 0 | 0 | 0 | 0 | 0 | 0 | 1 | 3 | 0 | 0 | 0 | 0 | 0 | 0 | 0 | 0 | 0 | 0 |
| Total rats | 237 | 349 | 81 | 82 | 69 | 68 | 45 | 39 | 13 | 14 | 48 | 37 | 24 | 25 | 61 | 33 | 24 | 23 | 37 | 30 |

FIG. 1.—Percentage of tumour-bearers and non-tumour-bearers at successive age periods.

bearers decreased to 34 per cent (i.e. 65 of 188 rats autopsied), 25 per cent, of rats in the 24–30 month age group were non-tumour-bearing and 12 per cent in the 30–36 month age group. While age tends to render the rat susceptible to tumours, the fact that tumours do not develop in all old rats suggests that some requirement other than that dependent only on the ageing process needs to be satisfied before a tumour will develop.

A total of 520 neoplasms was encountered in 379 tumour-bearing rats, the average number of tumours per rat being 1·3 for males and 1·4 for females. The

Fig. 2.—Distribution of all neoplasms according to age and sex.

majority of rats (266 of 379 or 70 per cent) bore one tumour, 23 per cent (78 of 379) two tumours, 6 per cent three tumours and less than 1 per cent four tumours.

The age and sex distribution of the 520 neoplasms are presented in Fig. 2. No tumour was observed in a rat under the age of 12 months. Two tumours were found in a total of 15 rats dying between the ages of 12 and 14 months. Thereafter the number of tumours encountered in each age group increased steadily to reach a maximum in the 26–28 month age group.

Nineteen varieties of benign and malignant tumours were observed implicating 13 different organs (Table III). In males, phaeochromocytoma, the most frequent neoplasm, was present in 62·8 per cent (137 of 218) of all rats examined. Interstitial cell tumours of the testis were detected in 18·4 per cent (42 of 228), carcinoma

TABLE III.—*Types, Order of Frequency and Percentage of Spontaneously-Occurring Neoplasms in GG Strain Rats Receiving the Basal Diet (Diet 1)*

Male

| Order of frequency of tumours | Number of tissues examined | Number of tumours | Percentage of tumours |
|---|---|---|---|
| Adrenal | 218 | 137 | 62·8 |
| Testis | 228 | 42 | 18·4 |
| Thyroid | 205 | 18 | 8·6 |
| Pituitary | 190 | 15 | 7·9 |
| Mediastinal | 232 | 14 | 6·0 |
| Pancreas : | | | |
| Carcinoma of islets | 207 | 1 | 4·3 |
| Adenoma of islets | | 8 (total 9) | |
| Kidney : | | | |
| Lipofibroma | 231 | 1 | 0·86 |
| Fibrosarcoma | | 1 (total 2) | |
| Liver sarcoma | 231 | 1 | 0·44 |

Female

| Order of frequency of tumours | Number of tissues examined | Number of tumours | Percentage of tumours |
|---|---|---|---|
| Adrenal | 268 | 127 | 47·4 |
| Breast | 316 | 57 | 18·0 |
| Pituitary | 316 | 40 | 12·7 |
| Thyroid | 221 | 25 | 11·3 |
| Mediastinal | 316 | 15 | 4·7 |
| Ovary : | | | |
| Thecal cell tumour | 216 | 1 | 2·3 |
| Mesonephroma | | 2 | |
| Granulosa cell tumour | | 1 | |
| Mesothelioma | | 1 (total 5) | |
| Liver sarcoma | 316 | 5 | 1·5 |
| Uterus : | | | |
| Fibromyoma | 219 | 2 | 1·8 |
| Carcinoma | | 2 (total 4) | |
| Pancreas : | | | |
| Adenoma of islets | 250 | 2 | 0·8 |
| Submaxillary carcinoma | 316 | 1 | 0·3 |
| Meningioma | 316 | 1 | 0·3 |

of the thyroid in 8·6 per cent, adenoma of the pituitary in 7·9 per cent, and mediastinal tumours in 6·0 per cent. In this connection, it should be mentioned that all tumours arising from the mesothelium of the pericardium and pleura and from the reticulo-endothelial cells of the thymus and lymph nodes in the hilus of the lung were classified as mediastinal neoplasms. Noteworthy was the presence of eight islet cell tumours of the pancreas and one carcinoma of the pancreas in a total of 207 glands examined histologically.

In females, as in males, phaeochromocytoma was the most frequent neoplasm and was identified in 127 of 268 rats (47·4 per cent). Fibroadenoma of the breast (18·0 per cent) was second in order of frequency, followed by pituitary adenoma (12·7 per cent) and thyroid carcinoma (11·3 per cent). All the other neoplasms, including islet cell adenoma, carcinoma and fibromyoma of the uterus, sarcoma of the liver, submaxillary carcinoma and meningioma were present in less than 5 per cent of rats. With the exception of the carcinoma of the submaxillary gland, no tumours were found associated with the digestive tract.

Phaeochromocytoma and islet cell tumours of the pancreas showed a higher frequency in males than in females while fibroadenoma of the breast was higher amongst the females. No other sex differences in tumour frequency were shown to be statistically significant.

The age distribution of each of the neoplasms is recorded in Table IV while histograms of some of the more commonly occurring neoplasms, namely, of the adrenal, breast, pituitary, thyroid and testis are shown in Fig. 3–7. Tumours of the anterior mediastinum and fibroadenoma of the breast were the earliest to make their appearance having been observed first in rats aged 12–14 months. Phaeochromocytoma and adenoma of the pituitary were first observed at 14–16 months although, as reported previously, phaeochromocytoma can occur at the age of 11 months (Gillman, Gilbert and Spence, 1953). Liver sarcoma was first

TABLE IV.—*Age and Sex Distribution of Different Types of Spontaneously-occurring Neoplasms in GG Strain Rats (Basal Diet)*

| Age group (months) | Adrenal RE. (M.) | Adrenal T. (M.) | Adrenal RE. (F.) | Adrenal T. (F.) | Thyroid RE. (M.) | Thyroid T. (M.) | Thyroid RE. (F.) | Thyroid T. (F.) | Pancreas (islet cell) RE. (M.) | Pancreas (islet cell) T. (M.) | Pancreas (islet cell) RE. (F.) | Pancreas (islet cell) T. (F.) | Mediastinal RE. (M.) | Mediastinal T. (M.) | Mediastinal RE. (F.) | Mediastinal T. (F.) |
|---|---|---|---|---|---|---|---|---|---|---|---|---|---|---|---|---|
| 2<4 | 0 | 0 | 0 | 0 | 0 | 0 | 0 | 0 | 0 | 0 | 0 | 0 | 0 | 0 | 0 | 0 |
| 4<6 | 1 | 0 | 0 | 0 | 1 | 0 | 0 | 0 | 1 | 0 | 0 | 0 | 1 | 0 | 0 | 0 |
| 6<8 | 0 | 0 | 9 | 0 | 0 | 0 | 0 | 0 | 0 | 0 | 9 | 0 | 0 | 0 | 9 | 0 |
| 8<10 | 1 | 0 | 7 | 0 | 1 | 0 | 0 | 0 | 1 | 0 | 7 | 0 | 1 | 0 | 7 | 0 |
| 10<12 | 2 | 0 | 17 | 0 | 2 | 0 | 14 | 0 | 2 | 0 | 14 | 0 | 2 | 0 | 17 | 0 |
| 12<14 | 1 | 0 | 7 | 0 | 2 | 0 | 5 | 0 | 2 | 0 | 9 | 0 | 2 | 0 | 12 | 2 |
| 14<16 | 3 | 2 | 20 | 8 | 2 | 0 | 12 | 0 | 1 | 0 | 17 | 0 | 3 | 0 | 23 | 0 |
| 16<18 | 7 | 3 | 18 | 5 | 6 | 0 | 15 | 0 | 6 | 0 | 18 | 0 | 7 | 0 | 23 | 2 |
| 18<20 | 20 | 10 | 21 | 7 | 19 | 0 | 11 | 0 | 18 | 0 | 21 | 0 | 21 | 2 | 23 | 2 |
| 20<22 | 33 | 19 | 30 | 15 | 33 | 2 | 23 | 1 | 26 | 0 | 29 | 1 | 33 | 2 | 34 | 1 |
| 22<24 | 36 | 19 | 33 | 19 | 38 | 0 | 30 | 4 | 37 | 0 | 30 | 0 | 39 | 1 | 38 | 1 |
| 24<26 | 37 | 26 | 36 | 14 | 30 | 3 | 30 | 2 | 35 | 5 | 34 | 0 | 41 | 1 | 43 | 3 |
| 26<28 | 55 | 39 | 41 | 24 | 50 | 11 | 45 | 9 | 55 | 3 | 46 | 0 | 57 | 5 | 54 | 3 |
| 28<30 | 22 | 17 | 26 | 14 | 22 | 2 | 23 | 2 | 23 | 1 | 20 | 0 | 26 | 2 | 27 | 1 |
| 30<32 | 4 | 2 | 24 | 14 | 3 | 0 | 20 | 4 | 4 | 0 | 18 | 1 | 3 | 1 | 25 | 0 |
| 32<34 | 0 | 0 | 9 | 5 | 0 | 0 | 7 | 3 | 0 | 0 | 5 | 0 | 0 | 0 | 11 | 0 |
| 34<36 | 0 | 0 | 3 | 2 | 0 | 0 | 0 | 0 | 0 | 0 | 3 | 0 | 0 | 0 | 3 | 0 |
| Total |  | 137 |  | 127 |  | 18 |  | 25 |  | 9 |  | 2 |  | 14 |  | 15 |

| Age group (months) | Pituitary RE. (M.) | Pituitary T. (M.) | Pituitary RE. (F.) | Pituitary T. (F.) | Breast RE. (M.) | Breast T. (M.) | Breast RE. (F.) | Breast T. (F.) | Testis RE. (M.) | Testis T. (M.) | Uterus RE. (F.) | Uterus T. (F.) | Ovary RE. (F.) | Ovary T. (F.) | Liver RE. (M.) | Liver T. (M.) | Liver RE. (F.) | Liver T. (F.) | Total tumours M. | Total tumours F. |
|---|---|---|---|---|---|---|---|---|---|---|---|---|---|---|---|---|---|---|---|---|
| 2<4 | 0 | 0 | 0 | 0 | 0 | 0 | 0 | 0 | 0 | 0 | 0 | 0 | 0 | 0 | 0 | 0 | 0 | 0 | 0 | 0 |
| 4<6 | 1 | 0 | 0 | 0 | 1 | 0 | 0 | 0 | 1 | 0 | — | — | — | — | 1 | 0 | 0 | 0 | 0 | 0 |
| 6<8 | 0 | 0 | 9 | 0 | 0 | 0 | 9 | 0 | — | — | 0 | 0 | 0 | 0 | 0 | 0 | 9 | 0 | 0 | 0 |
| 8<10 | 1 | 0 | 7 | 0 | 1 | 0 | 7 | 0 | 1 | 0 | 0 | 0 | 0 | 0 | 1 | 0 | 7 | 0 | 0 | 0 |
| 10<12 | 2 | 0 | 17 | 0 | 2 | 0 | 17 | 0 | 2 | 0 | 0 | 0 | 0 | 0 | 2 | 0 | 17 | 0 | 0 | 0 |
| 12<14 | 1 | 0 | 12 | 0 | 1 | 0 | 12 | 1 | 1 | 0 | 9 | 0 | 6 | 0 | 2 | 0 | 12 | 0 | 0 | 3 |
| 14<16 | 3 | 0 | 23 | 2 | 3 | 0 | 23 | 0 | 3 | 0 | 14 | 0 | 13 | 0 | 3 | 0 | 23 | 0 | 2 | 10 |
| 16<18 | 6 | 0 | 23 | 0 | 7 | 0 | 23 | 3 | 7 | 0 | 14 | 0 | 14 | 0 | 7 | 0 | 23 | 2 | 3 | 12 |
| 18<20 | 13 | 1 | 23 | 3 | 21 | 0 | 23 | 3 | 21 | 3 | 15 | 0 | 15 | 1 | 21 | 0 | 23 | 0 | 16 | 17 |
| 20<22 | 27 | 2 | 34 | 5 | 33 | 0 | 34 | 1 | 31 | 4 | 24 | 0 | 24 | 0 | 33 | 1 | 34 | 1 | 30 | 26 |
| 22<24 | 31 | 3 | 38 | 4 | 39 | 0 | 38 | 5 | 37 | 5 | 25 | 0 | 27 | 0 | 39 | 0 | 38 | 2 | 28 | 35 |
| 24<26 | 35 | 4 | 43 | 3 | 41 | 0 | 43 | 8 | 41 | 8 | 29 | 1* | 28 | 0 | 41 | 0 | 43 | 0 | 47 | 31 |
| 26<28 | 52 | 3 | 54 | 11 | 57 | 0 | 54 | 14 | 57 | 11 | 43 | 0 | 44 | 3 | 55 | 0 | 54 | 0 | 74 | 64 |
| 28<30 | 19 | 2 | 27 | 2 | 26 | 0 | 27 | 7 | 26 | 9 | 18 | 1† | 15 | 0 | 26 | 0 | 27 | 0 | 33 | 27 |
| 30<32 | 3 | 0 | 25 | 8 | 3 | 0 | 25 | 7 | 4 | 2 | 15 | 1† | 18 | 0 | 4 | 0 | 25 | 0 | 5 | 35 |
| 32<34 | 0 | 0 | 11 | 2 | 0 | 0 | 11 | 7 | 0 | 0 | 10 | 1* | 9 | 1 | 0 | 0 | 11 | 0 | 0 | 19 |
| 34<36 | 0 | 0 | 3 | 0 | 0 | 0 | 3 | 1 | 0 | 0 | 3 | 0 | 3 | 0 | 0 | 0 | 3 | 0 | 0 | 3 |
| Total |  | 15 |  | 40 |  | 0 |  | 57 |  | 42 |  | 4 |  | 5 |  | 1 |  | 5 | 238 | 282 |

RE. = rats examined.

T. = tumour.

* Myoma.

† Carcinoma.

*Other tumours :*

1 kidney lipofibroma 26 < 28 months.

1 fibrosarcoma of kidney pelvis (male) 26 < 28 months.

1 carcinoma submaxillary (female) 18 < 20 months.

1 meningioma (female) 20 < 22 months.

FIG. 3.—Distribution of 264 phaeochromocytoma according to age and sex.

FIG. 4.—Age distribution of 57 cases of fibroadenoma of the breast.

seen at 16–18 months, ovarian and testicular neoplasms at 18–20 months and islet cell tumours of the pancreas as well as thyroid carcinoma at 20–22 months.

From the foregoing, it is evident that (1) GG strain rats show a high frequency of spontaneous neoplasms, 74 per cent of all males and 58 per cent of all females

FIG. 5.—Distribution of 55 cases of pituitary adenoma according to age and sex.

FIG. 6.—Distribution of 43 cases of thyroid carcinoma according to age and sex.
FIG. 7.—Age distribution of 42 cases of interstitial cell tumours of the testis.

being tumour-bearers when more than a year of age ; (2) neoplasms of the endocrine glands predominate in both sexes with high frequencies of phaeochromocytoma and interstitial cell tumours of the testis in males and of phaeochromocytoma and fibroadenoma of the breast in females ; (3) with the exception of mediastinal neoplasms, sarcomata were rare and (4) no neoplasms of the respiratory tract or of the epithelium of the oesophagus, stomach and intestine were found in the series of 586 rats.

### II. *Effect of Diet on the Frequency Rates of Spontaneous Neoplasms*

In order to determine whether diet could modify the frequency rates of the spontaneous neoplasms described in the GG strain rats fed the basal ration of the colony (diet 1), a series of three diets was devised which, though fundamentally different from each other, were adequate to permit a steady increase in body weight and an expectation of life in accordance with that established for the GG strain in Johannesburg. Diet 2 (Table I) differed from the basal diet at least in two respects ; first, fibrin was used as the main source of animal protein in place of that derived from skimmed milk powder, and secondly, corn starch was substituted for wheat flour as a source of carbohydrate. In diet 3, carbohydrate was provided by potatoes, and animal protein by skimmed milk powder ; the amount of brewer's yeast was increased to 8 per cent (as compared with 2 per cent in diet 1) while Steenbock's salt mixture was excluded. Diet 4 contained no carbohydrate, except for the negligible amounts available in the yeast, while protein was supplied at the 77 per cent level mainly as casein. This diet could be regarded as a very high protein, carbohydrate-free, low fat diet. Diet 5 was the same as the basal diet (diet 1) except that the rats received a restricted amount of food from the time of weaning, namely, 5 g. per rat per day for the first 10 months after weaning and 7 g. per day for the remainder of their life-span. Diets 2 and 3 promoted a rate of growth as measured by body weight, similar to that of the control rats while rats receiving diets 4 and 5 showed a retarded growth rate, the average maximum weight achieved by the male rats, namely 334 g. and 264 g. respectively being significantly lower than the average maximum weight of the male controls (485 g.).

The age and sex distribution of all rats coming to autopsy in each of the above dietary groups is recorded in Table II and the percentage of rats alive at each age period in Table V. In all groups, at least 75 per cent and as many as 98 per cent of male and female rats were still alive at the age of 12 months and not less than 69 per cent (diet 3) at 18 months. In the 18–24 month age group, the mortality rate increased sharply with the result that with the exception of those receiving diet 5, the percentage of rats alive at 24 months did not exceed 69 per cent (diet 4, females) and fell as low as 37 per cent (diet 3, males). By contrast, 76 and 71 per cent of male and female rats respectively receiving diet 5 were still alive at 24 months. All rats receiving diets 1, 2, 3 and 4 were dead by the age of 36 months, whereas 15 per cent (1 out of 13) of males and 21 per cent (4 out of 14) of females receiving diet 5 survived beyond the age of 36 months but came to autopsy during the next 6-month period. Since statistical examination of the data revealed no significant differences in the expectation of life of rats receiving diets 1–5, the several dietary groups were regarded as comparable for purposes of analysis of tumour frequency rates.

*Tumour-bearing rats.*—The number of tumour-bearers, the total number of tumours and the rat : tumour ratio in rats receiving diets 1, 2, 3, 4 and 5 are listed in Table VI. The percentage of tumour-bearers was consistently lower in rats fed diets 2 and 3 and especially in diet 4 than in rats receiving the basal diet *ad libitum* (diet 1) or in restricted quantities (diet 5). Furthermore, the overall rat : tumour ratio was markedly reduced in rats receiving diet 4 (1 : 0·2 for males and 1 : 0·3 for females) but only slightly reduced in diets 2 and 3 as compared with control rats fed diet 1 (1 : 1·0 for males and 1 : 0·8 for females).

TABLE V.—*Expectation of Life in GG, Copenhagen and Utrecht Strains—Percentage of Rats Alive at Each Age Period*

| Age period | GG strain | | | | | | | | | | Copenhagen strain | | | | Utrecht strain | | | | | |
|---|---|---|---|---|---|---|---|---|---|---|---|---|---|---|---|---|---|---|---|---|
| | Basal diet (1) | | Diet 2 | | Diet 3 | | Diet 4 | | Diet 5 | | Parent | | 1st generation | | Parent | | 1st generation | | 2nd and later generations | |
| | M. (%) | F. (%) | M. (%) | F. (%) | M. (%) | F. (%) | M. (%) | F. (%) | M. (%) | F. (%) | M. (%) | F. (%) | M. (%) | F. (%) | M. (%) | F. (%) | M. (%) | F. (%) | M. (%) | F. (%) |
| 0 <6 | 99 | 100 | 100 | 91 | 97 | 94 | 97 | 100 | 100 | 100 | 91 | 95 | 100 | 92 | 100 | 94 | 100 | 100 | 97 | 96 |
| 6 <12 | 98 | 90 | 92 | 84 | 85 | 75 | 88 | 97 | 92 | 92 | 91 | 89 | 100 | 88 | 93 | 85 | 83 | 100 | 97 | 93 |
| 12 <18 | 93 | 73 | 80 | 73 | 69 | 69 | 71 | 97 | 84 | 85 | 85 | 84 | 100 | 84 | 74 | 57 | 75 | 87 | 89 | 76 |
| 18 <24 | 54 | 46 | 51 | 46 | 37 | 42 | 37 | 69 | 76 | 71 | 64 | 70 | 83 | 40 | 52 | 48 | 50 | 82 | 54 | 63 |
| 24 <30 | 16 | 11 | 11 | 17 | 4 | 1 | 11 | 15 | 38 | 50 | 19 | 22 | 33 | 16 | 18 | 18 | 29 | 35 | 16 | 26 |
| 30 <36 | 0 | 0 | 0 | 0 | 0 | 0 | 0 | 0 | 15 | 21 | 0 | 0 | 0 | 0 | 0 | 0 | 0 | 0 | 0 | 0 |
| 36 <42 | 0 | 0 | 0 | 0 | 0 | 0 | 0 | 0 | 0 | 0 | 0 | 0 | 0 | 0 | 0 | 0 | 0 | 0 | 0 | 0 |

TABLE VI.—*Showing Effect of Diet on the Number of Tumour Bearers and on Rat : Tumour Ratios in the GG Strain*

| Diet | Total rats | | Total tumour-bearers | | Percentage tumour-bearers | | Number of tumours | | Overall ratio of rats : tumours | | Ratio of tumour-bearers : tumours | | Actual number of rats with 1 or more tumours | | | | | | | |
|---|---|---|---|---|---|---|---|---|---|---|---|---|---|---|---|---|---|---|---|---|
| | | | | | | | | | | | | | 1 tumour | | 2 tumours | | 3 tumours | | 4 tumours | |
| | M. | F. | M. | F. | M. | F. | M. | F. | M. | F. | M. | F. | M. | F. | M. | F. | M. | F. | M. | F. |
| 1 | 237 | 349 | 175 | 204 | 74 | 58 | 238 | 282 | 1 : 1·0 | 1 : 0·8 | 1 : 1·3 | 1 : 1·4 | 120 | 146 | 47 | 40 | 8 | 16 | 0 | 2 |
| 2 | 81 | 82 | 47 | 34 | 58 | 41 | 61 | 47 | 1 : 0·7 | 1 : 0·6 | 1 : 1·3 | 1 : 1·4 | 38 | 25 | 6 | 5 | 1 | 4 | 2 | 0 |
| 3 | 69 | 68 | 38 | 24 | 56 | 35 | 45 | 28 | 1 : 0·6 | 1 : 0·4 | 1 : 1·1 | 1 : 1·2 | 32 | 20 | 6 | 4 | 0 | 0 | 0 | 0 |
| 4 | 45 | 39 | 10 | 12 | 22 | 31 | 11 | 13 | 1 : 0·2 | 1 : 0·3 | 1 : 1·1 | 1 : 1·1 | 9 | 11 | 1 | 1 | 0 | 0 | 0 | 0 |
| 5 | 13 | 14 | 9 | 8 | 69 | 57 | 10 | 12 | 1 : 0·8 | 1 : 0·8 | 1 : 1·1 | 1 : 1·4 | 8 | 4 | 1 | 4 | 0 | 0 | 0 | 0 |

As in diet 1, however, it was evident that (1) female tumour-bearers appeared to be less common than males and (2) more than 70 per cent of the tumour-bearers in each dietary group bore only one tumour. The percentage of rats carrying two tumours was highest in diet 5 (29 per cent) and lowest in diet 4 (9 per cent). The presence of 3 or of 4 neoplasms in a single rat was observed only in rats receiving diets 1 and 2 (Table VI).

From the foregoing, it is evident that diet 4 alone led to a significant reduction in the overall frequency of tumours as well as of tumour-bearing rats whereas diets 2 and 3 did not influence to any marked extent the tumour rates as compared with those observed in diet 1.

In general, it can be said that in all the dietary groups, as in the control, phaeochromocytoma was the most frequent neoplasm. However, rats fed diets 3, 4 and 5 differed from animals subsisting on diets 1 and 2 in that no sex difference was observed in the frequency of this neoplasm. The order and frequency of the other spontaneous neoplasms was variable although interstitial cell tumours of the testis and fibroadenoma of the breast in females tended to rank high in the sequence (Table VII).

By comparison with the basal diet, *diet* 2 (Table VII) led to a significant reduction in the frequency of phaeochromocytoma in both sexes, of fibroadenoma of the breast in females and of interstitial cell tumours of the testis in males. By contrast, adenoma of the pituitary in males and carcinoma of the uterus were significantly increased. Five cases of highly malignant carcinoma of the uterus were detected in a group of 58 females receiving diet 2 whereas only 2 cases of uterine carcinoma occurred in 219 female rats on the basal diet.

By comparison with the basal diet (diet 1), *diet* 3 (Table VII) promoted a significant reduction in the frequency of phaeochromocytoma in both sexes and of pituitary adenoma in females. Comparisons of the frequency of other spontaneous neoplasms disclosed no differences between rats fed diets 1 and 3.

*Diet* 4 (Table VII) resulted in a highly significant reduction in the frequency of phaeochromocytoma in both sexes, of fibroadenoma of the breast in females and a near significant reduction of pituitary adenoma in males as compared with rats fed diet 1. In comparison with diet 1, there was a tendency for tumour rates to be lower in rats receiving diet 4 even though the individual comparisons of tumour frequencies were not always significantly different.

When rats were fed restricted quantities of the basal diet (diet 5, Table VII), there were no significant differences in the frequencies of the neoplasms encountered as compared with rats receiving the same diet *ad libitum* (diet 1, Table I).

The differences in tumour frequency rates between rats fed diets 2, 3, 4 and those receiving the basal diet (diet 1) are summarized in Table VIII.

In Table IX attention is drawn to the first recorded appearance of each neoplasm in rats fed diets 2, 3, 4 and 5. As in the controls (diet 1), no tumour was observed under the age of one year. However, there was considerable individual variability in the time sequence of the various neoplasms. In diet 2, interstitial cell tumours of the testis which showed a low frequency were not observed before the age of 30 months as compared with 18 months in the rats fed diet 1 where the tumour rate was high. Similarly, the single case of fibroadenoma of the breast in rats receiving diet 4 was recorded in a rat aged 32 months whereas in the control rats (diet 1), this tumour was first seen at 12 months. By contrast, uterine carcinoma which showed an increased frequency in rats fed diet 2 appeared earlier (18 months)

than in the basal diet controls (24 months). The evidence suggests the existence of a possible relationship between the frequency of the neoplasm and the time of its appearance.

TABLE VII.—*Order of Frequency and Percentage of Tumours in All Necropsies of Rats Aged One Year and Over in GG Strain Rats Fed Different Diets*

**Diet 2**

| Male: Order of frequency of tumours | Number of organs examined | Number of tumours | Percentage of tumours | Female: Order of frequency of tumours | Number of organs examined | Number of tumours | Percentage of tumours |
|---|---|---|---|---|---|---|---|
| Adrenal | 66 | 31 | 47·0 | Adrenal | 62 | 18 | 29·0 |
| Thyroid | 67 | 11 | 16·4 | Pituitary | 69 | 9 | 13·0 |
| Pituitary | 74 | 12 | 16·0 | Thyroid | 57 | 5 | 8·8 |
| Testis | 72 | 2 | 2·7 | Uterus carcinoma | 58 | 5 | 8·6 |
| Pancreas adenoma of islets | 62 | 1 | 1·6 | Breast | 69 | 4 | 5·8 |
| | | | | Liver sarcoma | 68 | 3 | 4·4 |
| Breast | 74 | 1 | 1·3 | Pancreas adenoma of islets | 53 | 1 | 1·9 |
| Kidney fibrosarcoma | 74 | 1 | 1·3 | | | | |
| Liver sarcoma | 73 | 1 | 1·3 | Mediastinal | 69 | 1 | 1·4 |
| Mediastinal | 74 | 1 | 1·3 | Vagina carcinoma | 58 | 1 | 1·7 |
| Total | — | 61 | — | | — | 47 | — |

**Diet 3**

| Male | | | | Female | | | |
|---|---|---|---|---|---|---|---|
| Adrenal | 58 | 24 | 41·4 | Adrenal | 51 | 19 | 37·3 |
| Testis | 58 | 8 | 13·8 | Breast | 51 | 4 | 7·8 |
| Liver | 59 | 5 | 8·5 | Thyroid | 42 | 2 | 4·8 |
| Pituitary | 59 | 4 | 6·8 | Uterus carcinoma | 50 | 1 | 2·0 |
| Pancreas | 55 | 2 | 3·6 | Pituitary | 50 | 1 | 2·0 |
| Thyroid | 50 | 2 | 4·0 | Liver | 51 | 1 | 1·9 |
| Total | — | 45 | — | | — | 28 | — |

**Diet 4**

| Male | | | | Female | | | |
|---|---|---|---|---|---|---|---|
| Adrenal | 45 | 6 | 13·3 | Adrenal | 39 | 6 | 15·4 |
| Testis | 42 | 4 | 9·5 | Thyroid | 36 | 4 | 11·0 |
| Thyroid | 43 | 1 | 2·3 | Pituitary | 38 | 2 | 5·2 |
| | | | | Breast | 39 | 1 | 2·5 |
| Total | — | 11 | — | | — | 13 | — |

**Diet 5**

| Male | | | | Female | | | |
|---|---|---|---|---|---|---|---|
| Adrenal | 13 | 6 | — | Adrenal | 14 | 8 | — |
| Thyroid | 13 | 2 | — | Uterus carcinoma | 12 | 2 | — |
| Pituitary | 13 | 1 | — | Thyroid | 14 | 1 | — |
| Liver sarcoma | 13 | 1 | — | Liver sarcoma | 14 | 1 | — |
| Total | — | 10 | — | | — | 12 | — |

TABLE VIII.—*Effect of Diet on Tumour Rates as Compared with Tumour Rates in Rats Fed Basal Diet (Diet 1)*

| Diet | Tumour rate reduced | Tumour rate increased |
|---|---|---|
| 2 | Adrenal (male and female)<br>Breast (female)<br>Testis | Pituitary (male).<br>Uterus. |
| 3 | Adrenal (male)<br>Pituitary (female)<br>Mediastinal (male)<br>Liver (male) | *Nil.* |
| 4 | Adrenal (male and female)<br>Breast (female) | *Nil.* |

TABLE IX.—*Time of Appearance of Tumours*

| Age group (months) | GG strain Diet 1 | Diet 2 | Diet 3 | Diet 4 | Helsinki strain | Utrecht strain |
|---|---|---|---|---|---|---|
| 12 < 14 | Adrenal<br>Breast<br>Mediastinal | — | — | — | — | — |
| 14 < 16 | Pituitary | — | Adrenal<br>Liver sarcoma | — | Adrenal | Adrenal |
| 16 < 18 | Liver sarcoma | Liver sarcoma | — | — | Thyroid | — |
| 18 < 20 | Testis<br>Ovary | Adrenal<br>Breast<br>Uterus | Pituitary | Thyroid | — | Mediastinal |
| 20 < 22 | Thyroid<br>Pancreas | Thyroid<br>Pancreas<br>Pituitary<br>Ovary | Testis | Testis | Mediastinal<br>Pituitary<br>Testis | Thyroid<br>Liver sarcoma |
| 22 < 24 | — | — | — | Adrenal | Breast | Pituitary<br>Breast |
| 24 < 26 | Uterus | Mediastinal | Thyroid<br>Breast | Pituitary | Pancreas | Uterus |
| 26 < 28 | — | — | Pancreas<br>Uterus | — | — | Testis |
| 28 < 30 | — | — | — | — | Uterus | — |
| 30 < 32 | — | Testis | — | — | — | Pancreas |
| 32 < 34 | — | — | — | Breast | — | Ovary |

*Comment*

It is evident that diet can influence the frequency of tumours in rats of the same strain and living under similar environmental conditions (Table VIII). In view of the complexity of our experimental diets, it is not possible at this stage to attribute the frequency of any tumour to the presence in or absence from the diet of any particular factor. A cereal-free, high-protein, low-fat diet (diet 4), however, gave the lowest tumour frequency rates of all the experimental diets (Table VI). Although the rats fed this diet reached a maximum weight which was well below the maximum attained by the male controls (diet 1) nevertheless, as mentioned above, their life span was not significantly different from that of rats receiving diets 1, 2, 3 or 5.

It might well be thought that the reduction in tumour frequency in rats fed diet 4 was due to interference with growth as expressed by increments of weight especially as severe caloric restriction, accompanied by marked stunting decreased the incidence and delayed the appearance of spontaneous tumours (Saxton *et al.*, 1948) and delayed the appearance of some experimentally-induced tumours induced with known carcinogens (Rusch, Johnson and Kline, 1945 ; Tannenbaum, 1942). The decrease in the frequency of spontaneous tumours in the experiments reported by Saxton *et al.* was achieved by drastic retardation of growth in such a way that the body weight was maintained constant and was only allowed to increase by amounts of 5 g. at 50-day intervals. In these circumstances, the rats, after weaning, could not gain more than 100 g. during their life-time. While extreme retardation of body growth of the order described by Saxton *et al.* may significantly alter the frequency of spontaneous tumours, a moderate degree of retardation may have unpredictable effects on tumour frequency.

The fact that the tumour frequency was significantly reduced in rats fed diet 4 and unchanged in rats fed diet 5, despite a more severe retardation of growth in the latter series, suggests that it is not only the extent but the mechanism of production of retardation which will influence the frequency of spontaneous tumours. In the case of diet 4, the weekly increments in weight during the first 3 months were on an average 20 g. below those of the controls (diet 1). At this stage, the average weight of rats fed diet 4 was 252 g. in comparison with 290 g. for the controls. Thereafter, the weight curves diverged widely ; the diet 4 fed rats increased in weight at an extremely retarded rate to reach a maximum average of 334 g. (males) while the male controls gained weight rapidly to achieve their maximum weight of 485 g. at 13 months. The gross deficiency of carbohydrate in diet 4 obliged the rats to derive calories mainly from proteins and to a lesser extent from fat. The deamination of the proteins and the excessive outpouring of urea was accompanied throughout life by a polyuria and a greatly increased consumption of water.

In the case of diet 5, the daily ration of food was not adequate to satisfy the caloric requirements. As a consequence the rats were chronically hungry and consumed their ration voraciously within a few minutes after feeding. During the early period after weaning, gains in weight occurred very slowly and at 3 months, the average weight of the males (138 g.) was more than 100 g. lower than that of rats fed diet 4. By further slow increments of weight over a period of almost 2 years, the male rats receiving a restricted food intake achieved an average maximum weight of 264 g., that is to say, an average of 70 g. less than rats on diet 4 which lacked carbohydrates. Unlike the rats fed diet 4, the restricted diet rats (diet 5) never developed polyuria or polydipsia.

It is apparent that the metabolism emphasized by rats fed diet 4 differed markedly from that of rats subsisting on the restricted diet (diet 5) and was of a kind which obviously reduced the tumour frequency in one series (diet 4) despite the better growth performance, and not in the other (diet 5). However, the fact that it was possible to alter the frequency of tumours without producing any significant differences in growth performance indicates that the metabolism promoting tumours can be uncoupled from growth beyond certain limits.

The way diet mediates its effects on the tumour-promoting mechanism still remains to be determined. Whatever the effects of diet on metabolism, these are realized in large measure by an appropriate modification in endocrine physiology.

The high frequency of endocrine tumours in our experimental rats is more than suggestive evidence that diet had profoundly altered the functional and structural integrity of the endocrine glands. Tampering directly with the endocrine glands can prevent (Gillman, Gilbert and Spence, 1955), delay (Paschkis, Cantarow and Stasney, 1948; Bielschowsky and Hall, 1953), or even accelerate the emergence of neoplasms in response to specific carcinogens (Gillman *et al.*, unpublished data). It is not surprising, therefore, to find in our experiments that the frequency of fibroadenoma of the breast, carcinoma of the uterus, interstitial cell tumours of the testis was significantly modified by particular diets (Table VIII). This does not necessarily mean that the concentration of a particular carcinogen in the diet was increased or reduced; but it may signify that the diets created a suitable endocrine context for retarding or accelerating an underlying cancerization process which may have been determined in the first instance by factors other than diet. The influence of diet on the cancerization mechanism, therefore, may be at least three-fold namely, (1) through the presence of an anti-metabolite or carcinogen, (2) through its specific effects on the vitality of particular organs (imbalance, inadequacy or excess) and (3) indirectly through its influence on the physiology of the endocrine glands.

### III. *Comparison of the Frequency Rates of Spontaneous Neoplasms in Copenhagen, Utrecht and GG Strain Rats Reared in Johannesburg and Receiving the Same Diet*

The number of tumour-bearing rats and the number of tumours in the GG strain, Copenhagen, parent and first generation offspring and the Utrecht parent, first generation and second, third and fourth generation rats are presented in Table X. The percentage of tumour-bearing rats in the Copenhagen strain, parent as well as offspring, was higher both in males and in females while in the Utrecht strain it was consistently lower in males (parent and offspring) but higher in first generation, and in second, third and fourth generation females than in the GG strain.

The number of tumours per rat in the three strains under investigation was consistently higher in the Copenhagen strain, parent and offspring, than in the GG strain (Table X). In the Utrecht strain, this ratio was lower in males but higher in females as compared with the GG strain.

Examination of the data relating to the number of tumours per tumour-bearing rat disclosed that in the Copenhagen strain, unlike the GG strain, the percentage of rats with two tumours (35 per cent) was almost as great as that of rats with one tumour only (43 per cent). Furthermore, a considerable number of rats (17 per cent) bore three tumours while three rats carried four tumours (Table X). As a consequence, the number of tumours per tumour-bearing rat was higher in the Copenhagen strain (1 : 2·0 for males and 1 : 1·6 for females) than in the GG strain (1 : 1·3 for males and 1 : 1·4 for females). Similarly, in the Utrecht strain, where a relatively large percentage of females bore two or even three tumours, the number of tumours per tumour-bearing rat was consistently higher than in the GG strain females. With the exception of the first generation rats, the rat : tumour ratio in the *male* Utrecht strain was similar to that described for the GG strain males (Table X).

The first recorded appearance of the various neoplasms is presented in Table IX.

TABLE X.—*Tumour Bearers and Rat : Tumour Ratios in the GG, Copenhagen and Utrecht Strains Receiving the Same Diet (Diet 1)*

| Origin of strain | Total rats | | Total tumour-bearers | | Percentage tumour-bearers | | Number of tumours | | Overall ratio of rats : tumours | | Ratio of tumour-bearers : tumours | | Actual number of rats with 1 or more tumours: 1 tumour | | 2 tumours | | 3 tumours | | 4 tumours | |
|---|---|---|---|---|---|---|---|---|---|---|---|---|---|---|---|---|---|---|---|---|
| | M. | F. | M. | F. | M. | F. | M. | F. | M. | F. | M. | F. | M. | F. | M. | F. | M. | F. | M. | F. |
| *GG (Johannesburg)* | 237 | 349 | 175 | 204 | 74 | 58 | 238 | 282 | 1 : 1·0 | 1 : 0·8 | 1 : 1·3 | 1 : 1·4 | 120 | 146 | 47 | 40 | 8 | 16 | 0 | 2 |
| *Copenhagen*— | | | | | | | | | | | | | | | | | | | | |
| (Parent) . | 48 | 37 | 39 | 31 | 81 | 84 | 76 | 51 | 1 : 1·6 | 1 : 1·4 | 1 : 2·0 | 1 : 1·6 | 14 | 16 | 14 | 11 | 9 | 3 | 2 | 1 |
| (1st generation) | 24 | 25 | 23 | 18 | 96 | 72 | 38 | 26 | 1 : 1·6 | 1 : 1·0 | 1 : 1·6 | 1 : 1·4 | 11 | 10 | 12 | 8 | 1 | 0 | 0 | 0 |
| *Utrecht*— | | | | | | | | | | | | | | | | | | | | |
| (Parent) . | 61 | 33 | 37 | 18 | 60 | 54 | 47 | 29 | 1 : 0·8 | 1 : 0·9 | 1 : 1·3 | 1 : 1·6 | 29 | 11 | 6 | 3 | 2 | 4 | 0 | 0 |
| (1st generation) | 24 | 23 | 13 | 17 | 54 | 74 | 22 | 26 | 1 : 0·9 | 1 : 1·1 | 1 : 1·7 | 1 : 1·5 | 7 | 11 | 3 | 5 | 3 | 1 | 0 | 0 |
| (2nd, 3rd and 4th generation) | 37 | 30 | 24 | 21 | 65 | 70 | 31 | 40 | 1 : 0·8 | 1 : 1·3 | 1 : 1·3 | 1 : 1·9 | 16 | 8 | 6 | 9 | 2 | 3 | 0 | 1 |

1. (a) *Copenhagen migrant strain* (*parent*) (Table XI)

In addition to the tumours listed for the GG strain, reticulosarcoma of the mesenteric lymph node and a fibrosarcoma of the skin were encountered in the Copenhagen strain (Table XI). As in the GG strain, phaeochromocytoma was the most frequent tumour but, unlike the GG strain, there was no sex difference in the frequency of this tumour. Indeed, apart from fibroadenoma of the breast, no significant sex differences were established for any of the neoplasms encountered in the Copenhagen strain.

By comparison with the GG strain, the Copenhagen strain showed a significant increase in the frequency of phaeochromocytoma both in males and in females, of thyroid carcinoma and mediastinal neoplasms in males, of fibroadenoma of the breast in females and a suggestively near significant increase in pituitary adenoma

TABLE XI.—*Order of Frequency and Percentage of Tumours in All Necropsies of Rats Aged One Year and Over in the Copenhagen Strain*

Copenhagen strain (parent)

| Male: Order of frequency of tumours | Number of rats examined | Number of tumours | Percentage of tumours | Female: Order of frequency of tumours | Number of rats examined | Number of tumours | Percentage of tumours |
|---|---|---|---|---|---|---|---|
| Adrenal | 44 | 35 | 79·6 | Adrenal | 32 | 25 | 78·1 |
| Pituitary | 44 | 12 | 27·3 | Breast | 33 | 11 | 33·3 |
| Thyroid | 44 | 10 | 22·7 | Thyroid | 32 | 4 | 12·5 |
| Mediastinal | 44 | 10 | 22·7 | Pituitary | 33 | 4 | 12·1 |
| Testis | 42 | 3 | 7·1 | Mediastinal | 33 | 3 | 9·1 |
| Pancreas (Ca) | 44 | 3 | 6·8 | Uterus: | | | |
| Breast | 44 | 1 | 2·3 | Fibromyoma | 32 | 1 } 2 | 6·2 |
| Mesenteric lymph node | 44 | 1 | 2·3 | Myoma | | 1 | |
| | | | | Fibroendothelioma | 33 | 1 | 3·0 |
| Fibrosarcoma of skin | 44 | 1 | 2·3 | Ca vagina | 33 | 1 | 3·0 |
| Total | — | 76 | — | | — | 51 | — |

Copenhagen (1st generation offspring)

| Male: Order of frequency of tumours | Number of rats examined | Number of tumours | Percentage of tumours | Female: Order of frequency of tumours | Number of rats examined | Number of tumours | Percentage of tumours |
|---|---|---|---|---|---|---|---|
| Adrenal | 24 | 22 | 91·7 | Adrenal | 22 | 13 | 59·0 |
| Pituitary | 24 | 4 | 16·5 | Breast | 22 | 5 | 22·7 |
| Thyroid | 24 | 4 | 16·5 | Thyroid | 22 | 5 | 22·7 |
| Testis | 24 | 2 | 8·3 | Pancreas (adenoma of islets) | 21 | 1 | 4·7 |
| Pancreas (adenoma of islets) | 24 | 1 | 4·1 | | | | |
| | | | | *Liver | 22 | 1 | 4·5 |
| Breast | 24 | 1 | 4·1 | *Kidney | 22 | 1 | 4·5 |
| Stomach (Ca) | 24 | 1 | 4·1 | | | | |
| Olfactory | 24 | 1 | 4·1 | | | | |
| Sarcoma (root of tail) | 24 | 1 | 4·1 | | | | |
| Caecum (Ca) | 24 | 1 | 4·1 | | | | |
| Total | — | 38 | — | | — | 26 | — |

* Sarcoma.

in male rats (Table XII). Interstitial cell tumours of the testis were less common (bordering significance) in the Copenhagen than in the GG strain. With this exception, there was no reduction in the frequency of any neoplasm in the Copenhagen as compared with the GG strain.

(b) *Copenhagen strain, first generation offspring* (Table XI)

The kinds of tumours and the order of their frequency in the offspring did not differ markedly from those of the parent (Table XI). However, attention is drawn to (1) the development of a carcinoma of the stomach and to a carcinoma of the caecum, (2) the absence of mediastinal tumours from males as well as from females, (3) a significantly higher frequency of phaeochromocytoma in males than in females. It will be recalled that no sex difference in the frequency of phaeochromocytoma was established in the Copenhagen parent group.

A comparison of the frequency of individual neoplasms in the Copenhagen offspring with that of the parents disclosed only one difference, namely, a decrease in mediastinal tumours in male offspring rats. The Copenhagen first generation offspring, unlike the Copenhagen parent, when compared with the GG strain, showed differences only in respect of the phaeochromocytoma which occurred more frequently in the Copenhagen male offspring than in the GG strain male rats. The previously existing differences in respect of thyroid carcinoma, fibroadenoma of the breast and interstitial cell tumours of the testis were no longer in evidence in the Copenhagen first generation offspring (Table XII).

TABLE XII.—*Comparison of Tumour Frequency Rates in Copenhagen and Utrecht Strain with GG Strain*

| Diet | Tumour rate reduced | Tumour rate increased |
|---|---|---|
| Copenhagen (parent) | *Nil* | Adrenal (male and female).<br>Pituitary (male).<br>Thyroid (male).<br>Breast (female).<br>Mediastinal (male). |
| Copenhagen (1st generation offspring) | *Nil* | Adrenal (male). |
| Utrecht (parent) | Adrenal (male and female)<br>Testis | Mesenteric lymph node (male). |
| Utrecht (1st generation offspring) | Adrenal (female) | Thyroid (male and female).<br>Breast (female).<br>Pituitary (near significant female). |
| Utrecht (2nd, 3rd and 4th generation) | Adrenal (male and female) | Pituitary (male and female).<br>Breast (female). |

It can be concluded that the frequency rates of spontaneous neoplasms in the Copenhagen first generation offspring approached more closely to those of the GG strain than did the Copenhagen parents.

2. (a) *Utrecht migrant strain* (*parent*) (Table XIII)

The order of frequency of the spontaneous neoplasms is listed in Table XIII. Phaeochromocytoma was the most frequent neoplasm in males, while in females

TABLE XIII.—*Order of Frequency and Percentage of Tumours in All Necropsies of Rats Aged One Year and Over in Utrecht, Parent, 1st Generation and 2nd Generation Offspring*

Parent

Male

| Order of frequency of tumours | Number of tissues examined | Number of tumours | Percentage of tumours |
|---|---|---|---|
| Adrenal | 55 | 18 | 32·7 |
| Thyroid | 57 | 9 | 15·7 |
| Mesenteric* lymph node | 57 | 8 | 14·0 |
| Mediastinal | 57 | 3 | 5·2 |
| Pancreas (islet cell adenoma) | 50 | 2 | 4·0 |
| Testis | 55 | 2 | 3·6 |
| Liver sarcoma | 57 | 2 | 3·5 |
| Paraganglioma | 57 | 2 | 3·5 |
| Pituitary | 57 | 1 | 1·7 |
| Total | — | 47 | — |

Female

| Order of frequency of tumours | Number of tissues examined | Number of tumours | Percentage of tumours |
|---|---|---|---|
| Adrenal | 28 | 7 | 25·0 |
| Breast | 28 | 7 | 25·0 |
| Pituitary | 28 | 5 | 17·8 |
| Thyroid | 27 | 2 | 7·4 |
| Mesenteric* lymph node | 28 | 2 | 7·1 |
| Liver sarcoma | 28 | 2 | 7·1 |
| Uterus: (Leiomyosarcoma) (Myoma) | 24 | 1 | 8·3 |
| Pancreas | 27 | 1 | 3·7 |
| Ovary (thecal cell) | 26 | 1 | 3·8 |
| | — | 29 | — |

1st Generation offspring

Male

| Order of frequency of tumours | Number of tissues examined | Number of tumours | Percentage of tumours |
|---|---|---|---|
| Adrenal | 20 | 11 | 55·0 |
| Thyroid | 19 | 4 | 21·0 |
| Mediastinal | 20 | 3 | 15·0 |
| Breast | 20 | 1 | 5·0 |
| Testis | 20 | 1 | 5·0 |
| Mesenteric* lymph node | 20 | 1 | 5·0 |
| Liver sarcoma | 20 | 1 | 5·0 |
| Total | — | 22 | — |

Female

| Order of frequency of tumour | Number of tissues examined | Number of tumours | Percentage of tumours |
|---|---|---|---|
| Breast | 23 | 9 | 39·1 |
| Thyroid | 23 | 6 | 26·0 |
| Pituitary | 23 | 6 | 26·0 |
| Adrenal | 23 | 2 | 8·6 |
| Pancreas (islet cell adenoma) | 23 | 1 | 4·3 |
| Liver sarcoma | 23 | 1 | 4·3 |
| Sebaceous carcinoma of jaw | 23 | 1 | 4·3 |
| | — | 26 | — |

2nd generation offspring

Male

| Order of frequency of tumours | Number of tissues examined | Number of tumours | Percentage of tumours |
|---|---|---|---|
| Adrenal | 36 | 14 | 38·0 |
| Pituitary | 35 | 8 | 23·0 |
| Thyroid | 34 | 3 | 8·8 |
| Pancreas (islet cell adenoma) | 36 | 2 | 5·5 |
| Testis | 37 | 2 | 5·4 |
| Mediastinal | 37 | 1 | 2·7 |
| Liver sarcoma | 37 | 1 | 2·7 |
| Total | — | 31 | — |

Female

| Order of frequency of tumours | Number of tissues examined | Number of tumours | Percentage of tumours |
|---|---|---|---|
| Breast | 30 | 13 | 43·3 |
| Pituitary | 27 | 11 | 40·7 |
| Adrenal | 30 | 6 | 20·0 |
| Thyroid | 27 | 6 | 22·0 |
| Mediastinal | 30 | 3 | 10·0 |
| Pancreas (islet cell adenoma) | 30 | 1 | 3·3 |
| | — | 40 | — |

* Reticulosarcoma.

fibroadenoma of the breast occurred as commonly as phaeochromocytoma. Attention is also drawn to the higher frequency of mesenteric lymph node tumours (8 of 47) in male rats. With this latter exception, the kinds of tumours observed in the Utrecht strain were similar to those described in the GG strain. Sex differences in the frequency of neoplasms in the Utrecht strain were observed only in respect of pituitary adenoma which was higher in females than in males.

A comparison of the frequency rates of individual neoplasms in the Utrecht strain with that in the GG strain disclosed (1) a lower frequency of phaeochromocytoma in males and females, (2) a reduction in interstitial cell tumours of the testis, (3) a greater frequency of reticulosarcoma of the mesenteric lymph nodes in males and females, not a single case of this latter tumour having been reported in the GG strain (Table XII). It is noteworthy that reticulosarcoma comprised 13 per cent (10 of 76) of all neoplasms encountered in male and female rats of the Utrecht strain, thus ranking fourth in order of frequency.

(b) *Utrecht strain, first generation offspring* (Table XIII)

Amongst the females, the following features were noteworthy, namely, (1) fibroadenoma of the breast ranked first and constituted 39·1 per cent of all neoplasms and (2) phaeochromocytoma ranked fourth constituting only 8·6 per cent of all neoplasms (Table XIII). Amongst the male rats, attention is drawn to the absence of a pituitary adenoma and to the occurrence of only one case of reticulosarcoma of the mesenteric lymph node. A comparison of the sexes disclosed a higher frequency of phaeochromocytoma in males and of pituitary adenoma and fibroadenoma of the breast in females. In respect of the latter, the Utrecht offspring resembled the parent rats.

The frequency rates of neoplasms in male and in female rats of the Utrecht offspring did not differ significantly from that of the Utrecht parent. When the tumour frequency of the Utrecht offspring was compared with that of the GG strain, however, it was evident that (1) phaeochromocytoma was strikingly reduced in females, (2) fibroadenoma of the breast and carcinoma of the thyroid in females were increased and (3) no difference occurred in the frequency of neoplasms in male Utrecht offspring rats as compared with male GG strain rats (Table XII).

(c) *Utrecht strain, second, third and fourth generation offspring*

Since the frequency rates of spontaneous neoplasms in the Utrecht first generation offspring were not signficantly different from those of the Utrecht parent, it was decided to examine whether this pattern would be preserved in subsequent generations of these rats born and bred in our colony. Accordingly, a group of 67 rats, representing members of the second, third and fourth generation Utr cht offspring rats were grouped as a single series in order to compare the tumour frequency rate with that recorded (1) for the first generation offspring and (2) for the Utrecht parent (Table XIII).

The second, third and fourth generation rats differed in no respect from the first generation series although there was a tendency for pituitary adenoma in the males to occur more commonly than in the first generation males. When compared with the male Utrecht parent, the male second, third and fourth generation rats showed an increase in pituitary adenoma and a decrease in mesenteric lymph node tumours. No differences in tumour frequency rates were established for the females although there was a tendency for fibroadenoma of the breast and pituitary adenoma to be more common in the second, third and fourth generation rats than in the Utrecht parent.

A comparison of the second, third and fourth generation Utrecht rats with the GG strain revealed a markedly reduced frequency of phaeochromocytoma (males and females) and an increase in fibroadenoma of the breast (females) and in pituitary adenoma (males and females) (Table VIII).

From this experiment, it can be concluded first, that the Utrecht strain even when bred to the fourth generation, maintained a persistently lower frequency of phaeochromocytoma and a higher frequency of fibroadenoma of the breast than the GG strain and secondly, that the higher frequency of reticulosarcoma of the mesenteric lymph node in the migrant Utrecht strain was not maintained in the first and subsequent generations born and bred in Johannesburg.

*Comment*

No statistical data are available concerning the frequency rates of spontaneous tumours of Copenhagen strain rats reared in Europe. In the case of the Utrecht strain, data are available from Basle only in respect of phaeochromocytoma and fibroadenoma of the breast (*vide infra*). Apart from the tumours mentioned, therefore, it is not possible to say whether the tumour frequency in the Copenhagen and Utrecht strain rats, as observed in Johannesburg, had departed from that occurring in these same strains in Europe.

However, the kinds and the frequencies of the tumours observed in the Copenhagen and Utrecht strains differed in several respects from the tumour pattern in rats reported previously from Europe (Guérin, 1954) and from the United States (Curtis, Bullock and Dunning, 1931 ; Saxton *et al.*, 1948). Whereas 75 per cent of all tumours in the Copenhagen and 63 per cent in the Utrecht strain originated in the endocrine glands (adrenal, thyroid, pituitary, pancreas, ovary and testis), endocrine tumours accounted for 48, 40 and 2·2 per cent of all neoplasms in the series of rats described by Saxton *et al.*, Guérin and Curtis *et al.* respectively. Tumours of the adrenal medulla were greatly emphasized in the Copenhagen and Utrecht strains (47·2 and 32·9 per cent) but were rare in the rats described in the series of Guérin (1·5 per cent), of Saxton *et al.* (1·3 per cent) and of Curtis *et al.* (0·6 per cent) (Table XIV).

Reticulosarcoma of the mesenteric lymph node constituted 16·4 and 6·1 per cent of tumours encountered respectively by Curtis *et al.* and by Guérin, while Saxton *et al.* reported a high frequency of lymphosarcoma of the lung (40 per cent.). In the Copenhagen and Utrecht parent strains, reticulosarcoma of the mesenteric lymph node comprised 0·8 and 13·1 per cent respectively of all tumours whereas in the GG strain, not a single case of lymphosarcoma occurred in a total of 520 tumours (Table XIV).

In respect of the lymphosarcoma, the Utrecht migrant strain rats undoubtedly were akin to the rats of Curtis *et al.*, but this kinship was altered in the first generation Utrecht rats when the frequency of lymphosarcoma was sharply reduced to 2·2 per cent and to 0·0 per cent in the second and subsequent generations (Table XIII). In general, it can be said that the tumour pattern of the Copenhagen and Utrecht strains, and more particularly of the first and subsequent generation offspring, resembled more closely that of the GG strain than it did the tumour pattern in albino rats reported from Europe or from the United States

TABLE XIV.—*Comparison of Frequency Rates of Endocrine Tumours and of Lymphosarcoma in Different Groups of Rats*

| Type of tumour | Guérin (1954) | | Curtis *et al.* (1931) | | Saxton *et al.* (1948) | | Utrecht strain (Parent) Johannesburg | | Utrecht strain (1st generation) Johannesburg | | Copenhagen strain (Parent) Johannesburg | | GG strain Johannesburg | |
|---|---|---|---|---|---|---|---|---|---|---|---|---|---|---|
| | Number of tumours | Per-centage of all tumours | Number of tumours | Per-centage of all tumours | Number of tumours | Per centage of all tumours | Number of tumours | Per centage of all tumours | Number of tumours | Per-centage of all tumours | Number of tumours | Per-centage of all tumours | Number of tumours | Per-centage of all tumours |
| Adrenal . . | 9 | 1·5 | 3 | 0·6 | 2 | 1·3 | 25 | 32·9 | 13 | 27·1 | 60 | 47·2 | 264 | 50·7 |
| Thyroid . . | 89 | 15·5 | 0 | 0·0 | 0 | 0·0 | 11 | 14·4 | 10 | 20·8 | 14 | 11·0 | 43 | 8·3 |
| Pituitary . . | 93 | 16·2 | 0 | 0·0 | 69 | 46·0 | 6 | 7·9 | 6 | 12·5 | 16 | 12·6 | 55 | 10·5 |
| Pancreas . . | 1 | 0·2 | 0 | 0·0 | 0 | 0·0 | 3 | 3·9 | 1 | 2·1 | 3 | 2·4 | 11 | 2·1 |
| Ovary : | | | | | | | | | | | | | | |
| Benign . . | 28 | 5·7 | 6 | 1·8 | 1 | 0·6 | 1 | 1·3 | 0 | 0·0 | 0 | 0·0 | 5 | 0·9 |
| Malignant . | 5 | | | | | | | | | | | | | |
| Testis . . | 3 | 0·5 | 1 | 0·8 | 1 | 0·6 | 2 | 2·6 | 1 | 2·1 | 3 | 2·4 | 42 | 8·1 |
| Lymphosarcoma of mesenteric lymph node | 35 | 6·1 | 74 | 16·4 | 60* | 40·0 | 10 | 13·1 | 1 | 2·1 | 1 | 0·8 | 0 | 0·0 |
| Total number of spontaneous tumours observed | 567 | — | 452 | — | 150 | — | 76 | — | 48 | — | 127 | — | 520 | |

* Lymphosarcoma of lung in 59 cases.

We have already indicated from experiments with the GG strain that manipulation of the diet can alter the tumour frequency. It seems to be more than a coincidence, therefore, that the Copenhagen and Utrecht strains, reared in our laboratory in Johannesburg on the same diet fed to the GG strain for one or two generations should have developed a tumour pattern similar to that of the GG strain. Apart from diet, it should be mentioned that the Utrecht and Copenhagen rats were transported from sea level in Europe to an altitude of almost 6000 feet in the Southern Hemisphere. This sudden change in altitude and in geography in itself may have influenced profoundly the metabolism of the rats. It may well be that the influence of diet on tumorigenesis in Johannesburg, such as we have described in the GG, Copenhagen and Utrecht strains, may depend not only on the qualitative and quantitative attributes of the diet but also on the simultaneously modifying effect on metabolism of local geographical and climatic factors. Perhaps the diet which led to the emergence of a tumour pattern in the Copenhagen and Utrecht strains similar to that of the GG strain in Johannesburg might induce a different pattern of tumour frequency in these same strains of rats reared in other parts of the world where climatic and geophysical factors contrasted with those operating in Johannesburg. Furthermore, the possibility still needs to be excluded that the rats transferred from Europe to Johannesburg may have been isolated from a recurring source of infection operating in early life and causally related, for example to the emergence of reticulosarcoma.

On the basis of the data available at the present time, it is suggested that until these environmental factors have been fully characterized, it is not easily possible to assess the extent to which so-called strain differences in tumour frequency are attributable to one or more specific factors in the environment or to genetic factors or possibly to a modifying influence of both. The experiments conducted on the GG, Copenhagen and Utrecht strains as well as the reports from the literature raise the question as to the criteria to be used in assessing the relative importance of environmental and genetic factors in affecting biological reactions, including the susceptibility to various kinds of spontaneous tumours. The fact that the tumour frequency in the Copenhagen-first generation offspring tended to converge on a pattern similar to that recorded for the GG strain emphasizes the need for establishing the tumour frequency in rats under a variety of environments before it will be possible to know whether or not a particular tumour pattern is dependent on genetic or environmental factors.

### IV. *Effect of Change of Diet and Environment on the Frequency Rates of Phaeochromocytoma and of Fibroadenoma of the Breast in GG strain and Utrecht Strain Rats*

The primary object of this experiment was to determine whether the frequency of phaeochromocytoma in Utrecht strain rats reared in Basle would be modified by rearing the same strain of rat under different conditions of diet and of the physical environment in Johannesburg and similarly, to determine whether the frequency of this tumour would be altered in GG strain rats reared in Basle as compared with those reared in Johannesburg. Accordingly, at the time of weaning, a group of Utrecht strain litters in Basle and of GG strain rats in Johannesburg were divided equally, as far as possible, with regard to numbers and sex. One half of the GG strain rats were kept in Johannesburg and the other half transported to Basle. Likewise, a group of Utrecht strain weanling rats was kept in Basle and the litter mate controls sent to Johannesburg. In this way, it was hoped to compare the frequency of phaeochromocytoma in each of the two strains of rats under the conditions operating in Johannesburg and in Basle.

As the rats came to autopsy, all the organs, including the adrenals, of the GG strain and Utrecht strain rats kept in Johannesburg were systematically preserved for histological examination, in the event that, at a later stage, it might have been possible to compare the frequency of all spontaneous neoplasms. However, in the Utrecht and GG strain rats kept in Basle, only the adrenals and the breast were routinely examined histologically ; in the case of the other organs, a measure of selection was exercised in so far as only those organs which showed macroscopically pathological changes were preserved for microscopical study. As a consequence, for purposes of *statistical* analysis, it is possible in the present study to compare only the frequency of phaeochromocytoma and of fibroadenoma of the breast in the two groups of Utrecht strain and in the two groups of GG strain rats reared simultaneously in Johannesburg and Basle. However, useful information will become available relating to the kinds of spontaneous tumours in the Utrecht strain when reared in their European environment. The Utrecht rats reared in Johannesburg received the same diet as the GG strain, namely, diet 1 (Table I) while the GG strain transported to Basle were given access to the same diet* as the Utrecht strain kept in the Ciba laboratory, Basle.

The results of the four experiments are presented in Table XV. The frequency of phaeochromocytoma and of fibroadenoma of the breast in Utrecht strain rats reared in Basle did not differ significantly from that of the litter-mate migrants in Johannesburg. Moreover, as mentioned above, the tumour frequency in the first generation Utrecht offspring, born and bred in Johannesburg, did not differ from the migrating generation, the first significant change having been observed only in the second generation in respect of reticulosarcoma of the mesenteric lymph node. It would appear, therefore, that despite the profound change in diet and in the physical environment, the Utrecht strain migrant rats in Johannesburg preserved a frequency of phaeochromocytoma and of fibroadenoma of the breast similar to that of the Utrecht strain in Basle.

The GG strain migrant rats in Basle differed from the litter mate GG strain rats in Johannesburg in two ways. First, in the migrant group, the frequency of phaeochromocytoma, both in males and in females, was reduced and secondly, the previously reported sex difference in the frequency of phaeochromocytoma in GG strain rats in Johannesburg was no longer observed in the migrant GG strain reared in Basle. No changes were recorded in the frequency of fibroadenoma of the breast either in males or in females in the migrant group as compared with the litter mate controls in Johannesburg. It is thus apparent that the GG strain showed greater susceptibility to a change in the migrant generation than did the Utrecht strain. Moreover, it is noteworthy that the migrant GG strain rats, by showing a reduction in the frequency of phaeochromocytoma, were converging on a pattern similar to that observed in the Utrecht rats reared in Basle.

The modification in the frequency of phaeochromocytoma in the migrant GG strain rats could readily be attributed to diet alone since it was shown previously that manipulation of the diet (diets 2, 3 and 4, Table VIII) consistently led to a reduction of this tumour of GG strain rats in Johannesburg. No explana-

* Wayne " Lab-blox " (Rat diet) Allied Mills, Inc., Chicago, Ill.

TABLE XV.—*Table Showing the Frequency of Phaeochromocytoma and Breast Tumours in GG Strain and in Utrecht Strain Rats Reared in Johannesburg as well as in Basle*

| Type of tumour | GG strain | | | | | | | | Utrecht strain | | | | | | | |
|---|---|---|---|---|---|---|---|---|---|---|---|---|---|---|---|---|
| | Johannesburg | | | | Basle | | | | Johannesburg | | | | Basle | | | |
| | Female | | Male | | Female | | Male | | Female | | Male | | Female | | Male | |
| | RE. | Tum. | RE. | Tum. | RE. | Tum. | RE. | Tum. | RE. | Tum. | RE. | Tum. | RE. | Tum. | RE. | Tum. |
| Phaeochromocytoma | 274 | 122 | 218 | 136 | 34 | 7 | 32 | 9 | 33 | 7 | 59 | 16 | 27 | 2 | 43 | 9 |
| Breast tumours . | 316 | 57 | 231 | 0 | 47 | 10 | 49 | 1 | 28 | 7 | 57 | 0 | 32 | 11 | 63 | 3 |

RE. = Rats examined.
Tum. = Number of tumours.

tion can be offered for the failure of the tumours of the Utrecht migrant rats to be influenced by such contrasting environments as those operating in Johannesburg and in Basle. It might be thought that this stability of the Utrecht strain was genetically determined. However, it will be recalled that the frequency of reticulosarcoma of the mesenteric lymph node in the Utrecht migrant rats was sharply reduced in the first and second generation reared in Johannesburg. The fact that the frequency of one of the tumours was modified by environment suggests that (1) the frequency of phaeochromocytoma in the Utrecht strain is not necessarily genetically determined and (2) further manipulation of the diet and possibly other environmental factors may disclose a set of conditions whereby the frequency of phaeochromocytoma may be either increased or reduced, as happened in the GG strain following manipulation of the diet.

Apart from the phaeochromocytoma and fibroadenoma of the breast which were common both to the Utrecht and GG strains, it is noteworthy that the Utrecht rats reared in Basle developed the same kinds of tumours as those already described in the GG strain (Table XVI). Tumours of the endocrine glands were common and, amongst the males, phaeochromocytoma, as in the GG strain, ranked first in order of frequency. Amongst the females, however, phaeochromocytoma was observed in only 2 of 27 (7·4 per cent) rats examined. Nevertheless, both in males and in females, this tumour occurred far more frequently in the Utrecht strain than has been reported previously in albino rats reared in Europe (Guérin, 1954).

Fibroadenoma of the breast in the Utrecht strain was common in female rats ranking first in order of frequency in contrast to GG strain females in which phaeochromocytoma ranked first. Attention is also drawn to the occurrence of tumours of the mesenteric lymph nodes both in males and in females of the Utrecht strain in Basle in contrast to GG strain migrant rats in Basle which failed to develop this tumour. It will be recalled that lymphosarcoma of the mesenteric lymph node persisted in the Utrecht migrant rats reared in Johannesburg as well as in the first generation offspring, albeit in reduced numbers.

These experiments carried out in two geographically different regions emphasize the need for defining the relative participation of environmental factors in affecting the frequency of spontaneous neoplasms in a given strain of rat. It is also suggested that whereas profound changes in the environment may modify the frequency of some neoplasms within the migrant generation, these environmental factors may

TABLE XVI.—*Order of Frequency and Percentage of Tumours in All Necropsies on Rats Reared in Basle*

**Utrecht strain**

Male

| Order of frequency of tumours | Number of rats examined | Number of tumours | Percentage of tumours | *Minimum % of tumours |
|---|---|---|---|---|
| Pituitary | 28 | 8 | 28·9 | 12·9 |
| Adrenal | 43 | 9 | 20·8 | 14·5 |
| Pancreas | 22 | 4 | 18·1 | 6·4 |
| Thyroid | 28 | 5 | 17·9 | 8·1 |
| Testis | 32 | 2 | 6·2 | 3·2 |
| Lymph node | 20 | 1 | 5·0 | 1·6 |
| Breast | 65 | 3 | 4·6 | 4·8 |
| Gut | 24 | 1 | 4·1 | 1·6 |
| Liver | 41 | 1 | 2·1 | 1·6 |
| Lung | 43 | 6 | 1·4 | 9·6 |
| Stomach | — | 3 | — | 4·8 |
| Total | — | 43 | — | — |

* Expressed as percentage of total possible number of male rats over the age of 1 year, namely, 62.

Female

| Order of frequency of tumours | Number of rats examined | Number of tumours | Percentage of tumours | †Minimum % of tumours |
|---|---|---|---|---|
| Pituitary | 22 | 8 | 36·3 | 25·0 |
| Breast | 32 | 11 | 34·3 | 34·3 |
| Uterus | 22 | 4 | 18·1 | 12·5 |
| Lymph node | 16 | 3 | 18·7 | 9·3 |
| Pancreas | 17 | 3 | 17·6 | 9·3 |
| Thyroid | 19 | 2 | 10·5 | 6·2 |
| Ovary | 23 | 2 | 8·7 | 6·2 |
| Adrenal | 27 | 2 | 7·4 | 6·2 |
| Bladder | 13 | 1 | 7·6 | 3·1 |
| Lung | 28 | 1 | 3·5 | 3·1 |
| | — | 37 | — | — |

† Expressed as percentage of total possible number of female rats over the age of 1 year, namely, 32.

**GG strain**

Male

| Order of frequency of tumours | Number of rats examined | Number of tumours | Percentage of tumours | *Minimum % of tumours |
|---|---|---|---|---|
| Adrenal | 31 | 9 | 29·0 | 18·7 |
| Pituitary | 12 | 3 | 25·0 | 6·2 |
| Pancreas | 9 | 2 | 22·2 | 4·1 |
| Liver | 30 | 1 | 3·3 | 2·1 |
| Breast | 48 | 1 | 2·1 | 2·1 |
| Total | — | 16 | — | — |

* Expressed as percentage of total number of male rats over the age of 1 year, namely, 48.

Female

| Order of frequency of tumours | Number of rats examined | Number of tumours | Percentage of tumours | †Minimum % of tumours |
|---|---|---|---|---|
| Pituitary | 21 | 7 | 33·3 | 14·9 |
| Pancreas | 14 | 4 | 28·5 | 8·5 |
| Adrenal | 34 | 7 | 20·5 | 14·9 |
| Breast | 47 | 10 | 21·2 | 21·2 |
| Uterus | 15 | 1 | 6·6 | 2·1 |
| Thyroid | 16 | 1 | 6·2 | 2·1 |
| Lung | 33 | 1 | 3·0 | 2·1 |
| | — | 31 | — | — |

† Expressed as percentage of total possible number of female rats over the age of 1 year, namely, 47.

need to operate for a much longer period, that is, for at least one and possibly two or more generations before the frequency of other neoplasms will be significantly affected.

### SUMMARY

The results are reported of an investigation into the influence of diet and geography on the kinds of neoplasms and their frequency in three strains of albino rats.

A group of 586 GG strain rats reared in Johannesburg showed a high frequency of spontaneous neoplasms, 74 per cent of all males and 50 per cent of all females being tumour-bearers. Eighty per cent of all neoplasms were found in the endocrine glands, phaeochromocytoma being the commonest tumour in both sexes and interstitial cell tumours of the testis and fibroadenoma of the breast ranking second in order of frequency in males and females respectively.

By radical modification in the diet, without interference with life expectation, it was possible to achieve profound changes in tumour frequency rates including, amongst others, a reduction in phaeochromocytoma and an increase in the carcinoma of the uterus in GG strain rats. A high protein, carbohydrate-free diet reduced the overall frequency of tumours as well as of tumour-bearing rats. The modifications in the frequency of endocrine tumours suggested that the diets had created at least a favourable endocrine context for retarding or accelerating an underlying cancerization process which may have been determined in the first instance by factors other than diet.

A comparison of the frequency of spontaneous neoplasms in two migrant strains of albino rats, namely, Utrecht and Copenhagen, with that of the GG strain, when maintained under similar conditions of diet and environment, disclosed significant differences in respect of some neoplasms. Some of these differences were preserved in the first and subsequent generations of migrant strains whereas others were submerged with the result that the tumour frequency converged on a pattern similar to that of the GG strain. It was suggested that until the significant environmental factors, depending on diet and possibly also on the physical attributes of the environment, such as altitude, climate etc. were identified, it would not be possible to assess the extent to which so-called strain differences in tumour frequency could be attributed to one or more specific factors in the environment or to genetic factors.

We wish to record our appreciation to Dr. H. L. Stewart, National Cancer Institute, Bethesda, for his continued interest and encouragement.

We also wish to extend our sincere appreciation to Mrs. J. Bleloch for her invaluable assistance in the preparation of the statistical data, and to Miss J. van Veen for preparing the charts.

### REFERENCES

BERENBLUM, I.—(1954) "Carcinogenesis and tumour pathogenesis" *in* 'Advances in Cancer Research', **2**. Ed. Greenstein and Haddow (Academic Press Inc.), p. 129.

BIELSCHOWSKY, F. AND HALL, W. H.—(1953) *Brit. J. Cancer*, **7**, 358.

CURTIS, M. R., BULLOCK, F. D. AND DUNNING, W. F.—(1931) *Amer. J. Cancer*, **15**, 67.

GILLMAN, J. AND GILBERT, C.—(1954) *Ann. N.Y. Acad. Sci.*, **57**, 737.

*Iidem* AND SPENCE, I.—(1953) *Cancer*, **6**, 494.—(1955) *Experientia*, **11**, 158.

GUÉRIN, M.—(1954) 'Tumeurs spontanées des animaux de laboratoire'. Paris (Amédée legrand et Cie.).

MCCAY, C. H.—(1942) "Chemical aspects of ageing and the effect of diet upon ageing". Chap. 26 *in* 'Problems of Ageing'. Ed. E. V. Cowdry. Baltimore (Williams and Wilkins Co.).

PASCHKIS, K. R., CANTAROW, A. AND STASNEY, J.—(1948) *Cancer. Res.*, **8**, 257.

RUSCH, H. P.—(1944) *Physiol. Rev.*, **24**, 177.

*Idem*, JOHNSON, R. O. AND KLINE, B. E.—(1945) *Cancer Res.*, **5**, 705.

SAXTON, J. A., SPERLING, G. A., BARNES, LEROY, L. AND MCCAY, C. M.—(1948) *Acta Un. int. Cancr.*, **6**, 423.

TANNENBAUM, A. L.—(1942) *Cancer Res.*, **2**, 468.—(1944) *Ibid.*, **4**, 673.

YAMAGIWA, R. AND ITCHIKAWA, K.—(1914) *Verh. jap. path. Ges.*, cited by Woglom.

YEAKEL, E. H.—(1947) *Arch. Path.*, **44**, 71.